# Patterns of Ancestral Animal Codon Usage Bias Revealed through Holozoan Protists

**DOI:** 10.1093/molbev/msy157

**Published:** 2018-08-29

**Authors:** Jade Southworth, Paul Armitage, Brandon Fallon, Holly Dawson, Jarosław Bryk, Martin Carr

**Affiliations:** Department of Biological and Geographical Sciences, University of Huddersfield, Huddersfield, United Kingdom

**Keywords:** choanoflagellates, *Capsaspora*, optimal codons, premetazoan, translational accuracy, tRNA modification

## Abstract

Choanoflagellates and filastereans are the closest known single celled relatives of Metazoa within Holozoa and provide insight into how animals evolved from their unicellular ancestors. Codon usage bias has been extensively studied in metazoans, with both natural selection and mutation pressure playing important roles in different species. The disparate nature of metazoan codon usage patterns prevents the reconstruction of ancestral traits. However, traits conserved across holozoan protists highlight characteristics in the unicellular ancestors of Metazoa. Presented here are the patterns of codon usage in the choanoflagellates *Monosiga brevicollis* and *Salpingoeca rosetta*, as well as the filasterean *Capsaspora owczarzaki*. Codon usage is shown to be remarkably conserved. Highly biased genes preferentially use GC-ending codons, however there is limited evidence this is driven by local mutation pressure. The analyses presented provide strong evidence that natural selection, for both translational accuracy and efficiency, dominates codon usage bias in holozoan protists. In particular, the signature of selection for translational accuracy can be detected even in the most weakly biased genes. Biased codon usage is shown to have coevolved with the tRNA species, with optimal codons showing complementary binding to the highest copy number tRNA genes. Furthermore, tRNA modification is shown to be a common feature for amino acids with higher levels of degeneracy and highly biased genes show a strong preference for using modified tRNAs in translation. The translationally optimal codons defined here will be of benefit to future transgenics work in holozoan protists, as their use should maximise protein yields from edited transgenes.

## Introduction

The closest known relatives of metazoans consist of multiple lineages of unicellular eukaryotes known collectively as the holozoan protists. Within Holozoa the choanoflagellates are the sister-group to Metazoa (Carr et al. 2008), with the Filasterea being recovered as a more distantly related lineage (Shalchian-Tabrizi et al. 2008). The choanoflagellates are a large, diverse group of aquatic filter feeders, found in both freshwater and marine environments (see Leadbeater 2015 for a thorough review on the group). In contrast, only five species of filasterean have been described. Two of these are marine bacteriovores in the genus *Ministeria* (Patterson et al. 1993; Tong 1997) and two are predatory flagellates in the genus *Pigoraptor* (Hehenberger et al. 2017). The fifth species, *Capsaspora owczarzaki*, is a symbiont of the freshwater snail *Biomphalaria glabrata* (Stibbs et al. 1979; Hertel et al. 2002).

**Fig. 1.**
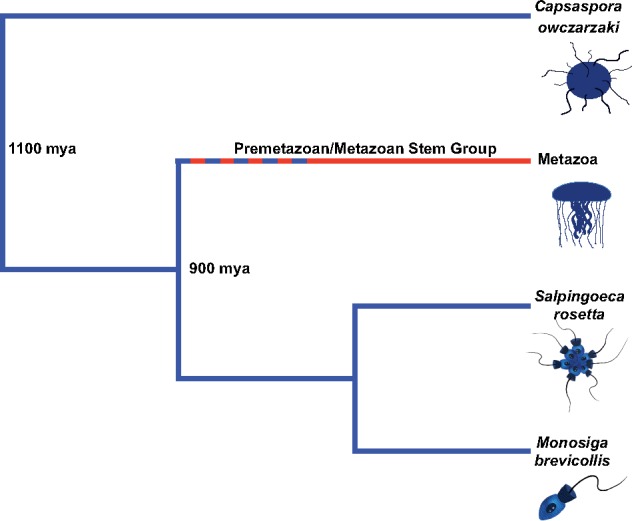
Simplified phylogeny of Holozoa. Lineages which show conserved ancestral traits are shown on blue branches. The red branch represents the loss of ancestral traits and the dotted blue/red line shows transitional stages during the process of loss. Approximate divergence dates are taken from Parfrey et al. (2011).

Studying their closest relatives has shone new light on the evolution of metazoans (Carr et al. 2010, 2017; Suga et al. 2013; Tucker 2013; Najile et al. 2016) and it is now clear that the last common ancestor of the holozoans had a complex genome, containing genes previously thought to be unique to Metazoa (Fairclough et al. 2013; Sebé-Pedrós et al. 2016; Hehenberger et al. 2017). Traits which are shared by both choanoflagellates and filastereans are candidates for being ancestral to the two groups. By extension such traits are also ancestral to Metazoa, even if the traits are not present in extant metazoans. Such traits must have been lost, either in a unicellular premetazoan or stem-group metazoan (fig. 1).

Whole genome sequences from the choanoflagellates *Monosiga brevicollis* (King et al. 2008) and *Salpingoeca rosetta* (Fairclough et al. 2013), as well as *C. owczarzaki* (Suga et al. 2013), have highlighted extensive gene loss and gain within holozoans; however, such studies have not examined the genomes on a population genetics level in order to determine the role of natural selection in their evolution. One study that has considered aspects of the population biology of choanoflagellates was that of Carr et al. (2017), which analyzed transcriptome sequences of 19 species of choanoflagellate to highlight variation in the strength of natural selection in the elongation factor genes EF-1A and EFL. The study used rates of nucleotide substitutions at synonymous and nonsynonymous sites (Ka/Ks) and also codon usage analyses to examine varying selective constraint across species.

The genetic code shows redundancy and Clarke (1970) speculated that there may be numerous selective forces controlling the codon preference employed by genes. Grantham et al. (1980) showed that codons are indeed used in a nonrandom fashion within eukaryotes, prokaryotes and viruses. The level of codon usage bias in a gene is commonly determined using the “effective number of codons” or *Nc* (Wright 1990). Values of *Nc* range from 20, in genes where each amino acid only uses a single codon, to 61, when codons are used equally for each amino acid. Numerous subsequent studies have shown that both natural selection and mutational pressures can shape codon usage (Sharp et al. 1995; Smith and Eyre-Walker 2001; Figuet et al. 2014). For those species where codon usage is driven by natural selection, it is assumed that they possess a suite of translationally “optimal codons” that provide a selective advantage when used in preference to nonoptimal codons. As originally defined (Ikemura 1981), optimal codons are those codons that complement the most abundant isoaccepting tRNA. Lloyd and Sharp (1991, 1993) proposed an alternative strategy to determine optimal codons, identifying those codons whose frequency of usage is significantly higher in putatively highly expressed genes. A third methodology, as implemented by the program CodonW (Peden, 1999) when expression data are absent, is to identify those codons are at significantly higher frequencies in the most biased genes, based upon axis 1 of a correspondence analysis, compared with the least biased genes in a genome. Peden (1999) noted that codons identified as optimal by CodonW can be considered to be genuine if the major trend in codon usage is for optimal translation and that highly biased genes are highly expressed. The usage of optimal codons in a gene can be described using *F*_op_, the Frequency of Optimal Codons, (Ikemura 1981), calculated by dividing the number of optimal codons present in a gene by the total number of codons.

Selection on the use of optimal codons may operate through two, not mutually exclusive, mechanisms. Codon usage bias is often more extreme in highly expressed genes (Gouy and Gautier 1982) and optimal codons may facilitate more rapid translation than nonoptimal codons (Pedersen 1984; Sørensen and Pedersen 1991). This led to the view that selection for optimal codons may be a result of selection for translational efficiency (Ehrenberg and Kurland 1984). In unicellular organisms, the rate of protein synthesis can have a profound effect on growth rate (Bulmer 1991), and therefore reproductive success, highlighting the importance of selection upon translational efficiency in protists. Proteins that contain misincorporated amino acids will constitute a metabolic burden, and therefore, selection upon codon usage may also reflect the requirement for accurately translated polypeptides. Consistent with this view, Precup and Parker (1987) showed that favored codons in *Escherichia coli* could lead to a 10-fold reduction in amino acid misincorporation compared with nonfavored codons. Akashi (1994) provided further evidence for selection acting upon translational accuracy by showing that preferred codons were enriched in regions encoding DNA-binding domains compared with nondomain regions in *Drosophila* transcription factors.

Selection coefficients (*s*) associated with the use of optimal codons over nonoptimal ones are estimated to be small, with *s *=* *1 × 10^−6^ to 1 × 10^−8^ in *Drosophila* (Hartl et al. 1994; Akashi 1995; Maside et al. 2004). Therefore, for selection to operate on synonymous codons, a species must possess a large effective population size (*N_e_*). Effective population sizes are currently unknown for choanoflagellate and filasterean species. Evidence from other unicellular eukaryotes indicates a broad range of values of *N_e_*, with effective population sizes in some taxa being similar to multicellular organisms but 2–4 orders of magnitude higher in other species (Snoke et al. 2006; Watts et al. 2013). Lynch and Conery (2003) argued that the transition from a unicellular existence to multicellularity in eukaryotes resulted in order-of-magnitude reductions in *N_e_*, indicating that selection could play a stronger role in codon usage bias in holozoan protists than in metazoans.

In the absence of efficient natural selection, codon usage is likely to be determined by mutational forces. Mutation pressures which are strong enough to bias neutral synonymous codon positions are also likely to influence nucleotide composition at noncoding sites such as introns and intergenic regions. A strong relationship between the GC-content at silent third positions (GC3s) and local base composition may indicate that mutational pressure is a major driver of codon usage; the absence of such a relationship would suggest that mutation does not play a strong role and points to natural selection as being the major player in codon choice (Kliman and Hey 1994).

The Carr et al. (2017) choanoflagellate study showed that genes encoding elongation factors, which are highly expressed (Liu et al. 1996), exhibited strong codon usage bias. This finding is consistent with selection on codon usage within choanoflagellates and promotes further investigation into the driving forces behind codon usage bias in holozoan protists. Some choanoflagellate species have intercontinental oceanic distributions (Carr et al. 2008, 2017; Nitsche et al. 2011), raising the possibility that some choanoflagellate taxa may possess very large population sizes. In contrast, *C. owczarzaki* is a symbiont, or parasite, of the snail *Biomphalaria glabrata*, and therefore, may undergo regular bottlenecks as it passes from one host to another; the result of such bottlenecks may act to suppress the effective population size of *C. owczarzaki*. A prediction of this symbiotic lifestyle is that *C. owczarzaki* may show less efficient selection operating upon its codon usage compared with free-living choanoflagellates.

The study presented here aims to investigate the forces that control codon usage bias within the three holozoan protists that currently have available whole genome sequences. General trends of codon usage and optimal codons identified have been determined for each species. Furthermore, the roles of mutational pressure and selection, on both translational efficiency and accuracy, were considered.

## Results

### Strength and Direction of Codon Usage Bias in the Holozoan Protists

The degree of codon usage bias was determined for all genes in the transcriptomes of *M. brevicollis*, *S. rosetta* and *C. owczarzaki* using the effective number of codons (*Nc*). Across the three species, values of *Nc* ranged from 20 to 61 and the mean transcriptome values are shown in table 1. *S. rosetta* shows the strongest codon usage bias across its transcriptome, whereas *M. brevicollis* and *C. owczarzaki* have similar, and higher, mean values of *Nc*.

**Table 1.** Whole Genome and Codon Usage Statistics in the Transcriptomes of the Three Holozoan Protists.

Species	Genome Size (Mb)^a^	Number of CDS Sequences	CDS % Genome^a^	GC-Content	GC3s (±SD)	*Nc* (±SD)	*F*_op_ (±SD)	*Ŝ*
*M. brevicollis*	41.6	9,171	39.7	0.549	0.638 ± 0.060	48.05 ± 5.62	0.572 ± 0.080	1.27
*S. rosetta*	55.0	11,736	43.5	0.566	0.707 ± 0.073	44.78 ± 5.36	0.576 ± 0.079	2.00
*C. owczarzaki*	28.0	10,123	58.7	0.538	0.653 ± 0.075	47.60 ± 6.45	0.494 ± 0.100	2.18

Note.—Mb, Megabases; CDS, Coding Sequence; GC3s, guanine+cytosine content at synonymous third positions; *Nc*, the effective number of codons; *F*_op_, the frequency of optimal codons; *Ŝ*, the strength of selected codon usage bias.

a Data taken from Fairclough et al. (2013) and Suga et al. (2013).

**Fig. 2.**
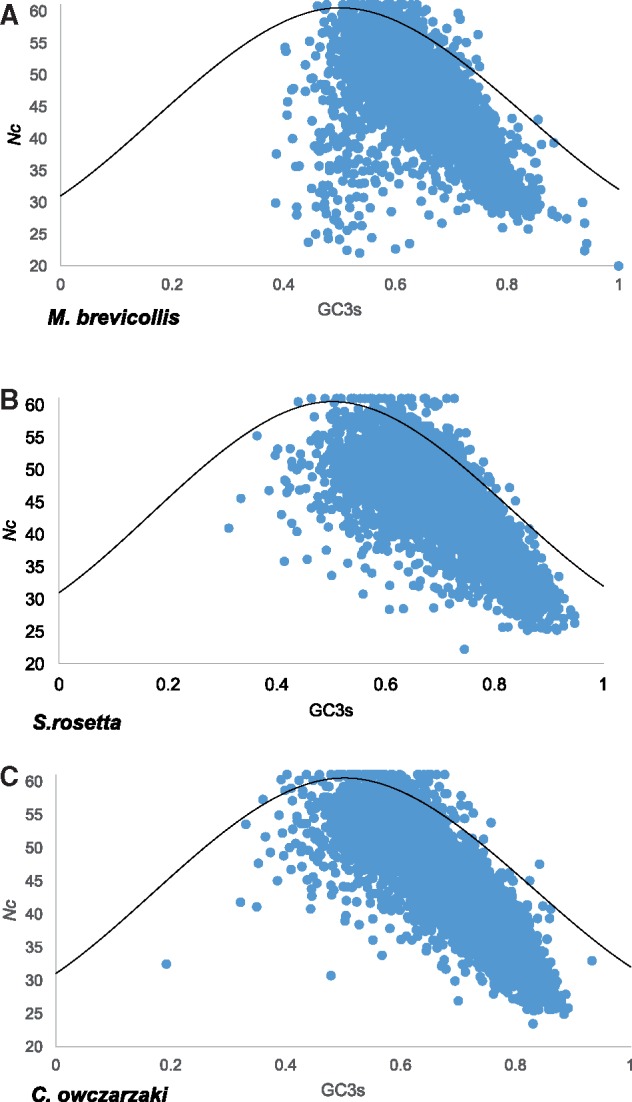
*Nc* plots for *M. brevicollis*, *S. rosetta* and *C. owczarzaki*. GC3s values are shown on the *x*-axis and *Nc* values are given on the *y*-axis. The curved line on each plot represents the expected position of genes evolving under a neutral mutation model (Wright 1990).

The proportion of GC at synonymous third positions (GC3s) highlights the direction of bias, toward GC or AT-ending codons. Figure 2 shows genes in all three species exhibit a strong GC bias, with highly biased genes showing higher GC3s. Consistent with their weaker codon usage bias, mean GC3s is lower in both *M. brevicollis* and *C. owczarzaki* than *S. rosetta* (table 1). Genes enriched for AT-ending codons are in a minority in all three species, with only 1.3%, 0.6%, and 1.5% of genes in *M. brevicollis*, *S. rosetta*, and *C. owczarzaki*, respectively, showing GC3s lower than 0.5.

The *Nc* plot of *M. brevicollis* differed from those of *S. rosetta* and *C. owczarzaki* due to the presence of approximately 200 genes which did not conform to the general trend of increasing GC3s with increasing bias. These genes were investigated further and most were shown to be 1) highly repetitive in their conceptual amino acid sequence, 2) lacking identifiable functional domains, 3) lacking identifiable orthologues through BLAST in either *S. rosetta* or *C. owczarzaki*, and 4) unable to be recovered when reciprocal BLASTs were performed with their top BLASTn hits (supplementary fig. S1, Supplementary Material online). Of the 200 genes examined, 159 could not be recovered with reciprocal BLAST analyses and only eight did not meet any of the four criteria above. This evidence points to many of these genes being false positive annotations rather than genuine genes. Excluding the 192 potential false positives has limited impact on the overall genome statistics for *M. brevicollis* (mean *Nc* = 48.28 ± 5.356, mean GC3s = 0.641 ± 0.057). As there is a lack of empirical evidence to confirm the genes as false positives, they have been retained in subsequent analyses with the proviso that they may be introducing noise into the results for *M. brevicollis*.

For species, the 5% highest, lowest and mid-biased genes were identified and used to study patterns of codon usage and base composition across the transcriptome. Within the *M. brevicollis* genome 75% of genes have been assigned KOG ontology functional groups and gene categories (King et al. 2008), allowing a comparison of ontology categories across codon usage bias categories (supplementary table S1, Supplementary Material online). The composition of three of the four functional KOG groups differed between the high bias genes and the other two bias categories. Genes involved in information storage and processing, as well as metabolism were significantly enriched in the highly biased genes (Fisher’s exact tests, *P < *0.02), whereas there was a paucity of poorly characterized genes in the high bias genes. Of the 25 KOG gene categories, four were significantly underrepresented in the high codon bias genes compared with the mid and low bias genes (*P *<* *0.005 in all four categories). Two of the underrepresented categories were for either general function, or function unknown genes; however, there was also a dearth of genes involved in signal transduction, as well as DNA replication and recombination, in the highly biased genes (*P *<* *0.005). Genes involved in protein translation made up the highest proportion of highly biased genes and this KOG category was significantly enriched in the high bias genes (*P *<* *0.0001). Two further categories, containing genes involved in energy production, as well as amino acid metabolism, were also enriched (*P *<* *0.02 in both KOG categories).

Although all three holozoans exhibit a strong bias towards GC-ending codons it is important to determine the evolutionary pressures that drive synonymous codons towards guanine and cytosine. It is therefore also important to determine whether, if selection is indeed operating upon synonymous codon usage, selection is operating at similar levels of efficiency across the three species.

### The Role of Local Mutation Pressure in Determining Codon Usage

One possible explanation for the relationship between *Nc* and GC3s is that codon usage is driven mainly by mutation pressure. Under the mutational model, such a bias toward GC in highly biased genes is likely to affect all neutral nucleotide positions within a given gene. In order to determine if mutation pressure is driving the high GC3s observed in highly biased genes, values of mean GC3s, as well as GC content in introns and flanking DNA were determined for the genes within the three categories of bias (supplementary table S2, Supplementary Material online).

**Fig. 3.**
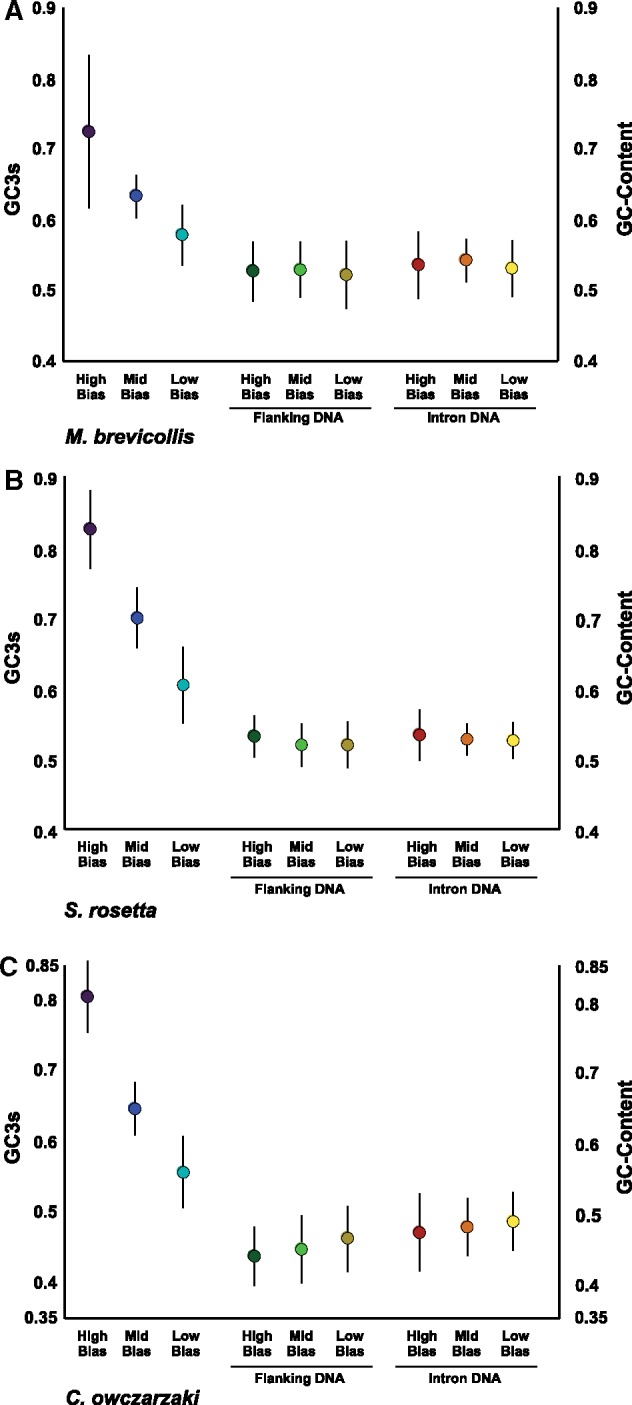
Comparisons of mean GC3s and noncoding DNA GC-content for the holozoan protists. The bars represent the standard deviation of the values from each category. The GC3s values are on the left *y*-axis (purple dot, highly biased; dark blue, mid bias; light blue, low bias). Noncoding GC content is shown on the right *y*-axis axis (Flanking DNA. Dark green dot, highly biased; light green, mid bias; mustard, low bias. Intron DNA. Red dot, highly biased; orange, mid bias; yellow, low bias).

Figure 3 shows that for each species GC3s decreases from the highly biased to medium and least biased genes, with significant differences in GC3s observed between categories for all species (*t* test, *P *<* *0.0001 in each comparison, supplementary table S2, Supplementary Material online). A *t* test could not be performed on the high and mid categories for *M. brevicollis*, as the putative erroneously annotated genes, which have relatively lower GC3s, produced a nonnormal distribution for this category (supplementary fig. S2, Supplementary Material online). In contrast, GC content produces very different patterns between all bias categories for noncoding DNA (both flanking DNA and introns) in each of the three species (supplementary table S2, Supplementary Material online). Only *S. rosetta* shows decreasing GC content as bias level decreases in noncoding DNA, which would be expected if mutation pressure was driving the high GC3s observed in the high bias gene categories in all three species. Within this species however there is no significant difference in GC content between mid and low bias genes for either flanking DNA or introns, in contrast to the significant difference in GC3s. *M. brevicollis* shows the highest noncoding GC content in mid bias genes, whereas *C. owczarzaki* shows the exact opposite pattern to GC3s, with the highest noncoding GC content observed in low bias genes and the lowest GC content recovered in the high bias genes (fig. 3 and supplementary table S2, Supplementary Material online). The GC content flanking DNA and introns in each bias category show similar patterns in each species, suggesting mutation patterns are similar across individual genes. Plotting GC3s against the GC content of introns failed to show any relationship between the two statistics (graphs not shown), with *R^2^* values of 0.004, 0.007, and 0.018 given for *M. brevicollis*, *S. rosetta*, and *C. owczarzaki*, respectively.

The stop codons of all three species also fail to show evidence for a GC-bias in mutation pressure. The highly biased genes, based upon *Nc*, in each species show a preference for the GC-free UAA stop codon over both guanine containing stop codons (supplementary table S3, Supplementary Material online). Following on from these findings, it appears that a mutational pressure towards guanine and cytosine is not a major driver of the variation observed in GC3s.

### Identification of Optimal Codons and Major tRNA Genes

The findings above suggest that natural selection for optimal codons is therefore an important force in determining codon choice. Optimal codons for all three species were determined using CodonW. In addition, optimal codons were also identified by comparing the 5% most highly and weakly expressed genes in *S. rosetta* and *C. owczarzaki*. The two strategies differed by two optimal codons for *S. rosetta* and one optimal codon for *C. owczarzaki*, indicating that the CodonW estimated optimal codons for *M. brevicollis* are likely to be accurate (table 2).

**Table 2.** Optimal Codons Designated for the Three Species of Holozoan Protist.

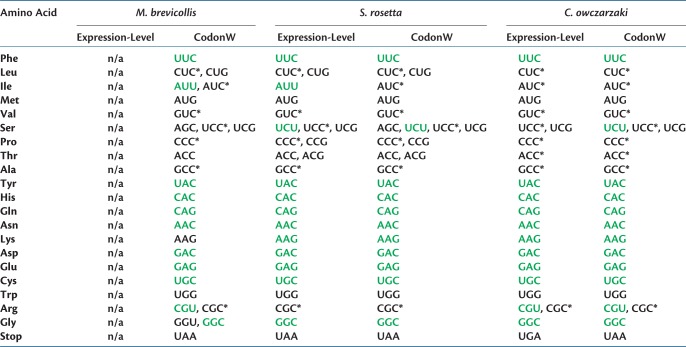

Note.—Optimal codons which complement the major tRNA gene are written in green. An asterisk denotes an optimal codon which would complement the major tRNA gene if the adenosine at the wobble position underwent deamination modification.

Consistent with the major bias toward GC-ending codons across the genome, 63 out of the 69 CodonW-estimated optimal codons across the three species are GC-ending; despite this, each species also possesses at least one optimal codon ending in uracil. In every case, uracil ending codons are optimal for 3-fold, 4-fold, or 6-fold degenerate amino acids and are also the least frequently used optimal codon for the encoded amino acid (supplementary table S3, Supplementary Material online). No optimal codons in any of the species possess adenine at synonymous sites. Eleven of the 18 amino acids showing redundancy have identical optimal codons across all three species (table 2). The remaining seven amino acids exhibiting redundancy all share at least one optimal codon, showing that the suite of optimal codons for all three species are remarkably similar.

tRNA genes were identified in each species, in order to compare optimal codons and major tRNA genes (either the most abundant or only tRNA gene for a given amino acid). The number and range of tRNA genes are also very similar across the three species. The number of identified tRNA genes ranges from 104 to 114 (supplementary table S4, Supplementary Material online), with *C. owczarzaki* harboring a lower number than either choanoflagellate. It can also be seen that the numbers of tRNA genes for each amino acid are also very similar, with only four out of 20 amino acids varying by >2 tRNA gene copies across the three species (supplementary table S4, Supplementary Material online).

With the exception of lysine in *M. brevicollis*, which does not possess a single most abundant tRNA gene, there was a perfect match between optimal codons and major tRNA genes in all 2-fold-degenerate amino acids (table 2). All three species have GGC as an optimal codon for glycine and also have the complementary GCC anticodon in their most abundant glycine tRNA genes (table 2). In contrast to this, for the remaining eight 3-fold to 6-fold-degenerate amino acids there were only six matches between optimal codons and major tRNA genes across the three species (table 2). For those six matches, the optimal codons ended in uracil, however the encoded amino acids showed a greater preference for GC-ending optimal codons in highly biased genes (supplementary table S3, Supplementary Material online). In the 3-fold to 6-fold-degenerate amino acids, most of major tRNA genes (22 out of 24) harbor adenine at the anticodon site complementary to the synonymous codon position. This suggests that the codons may rely upon the wobble effect for binding to their corresponding tRNA molecule. However, deamination of the adenine base in the anticodon, at the wobble position, to inosine will allow complementary base pairing to the cytosine nucleotides of optimal codons.

In the higher degeneracy amino acids, there is a complete absence of tRNA genes that perfectly complement cytosine-ending optimal codons (supplementary table S5, Supplementary Material online). Direct evidence for tRNA modification and deamination of adenine at the wobble position to inosine was provided by screening publicly available SRA transcriptome data sets. During reverse transcription inosine in modified RNA is replaced with guanine in cDNA molecules (Suzuki et al. 2015), therefore transcripts with adenine and guanine at the wobble position were screened for the 3-fold to 6-fold-degenerate amino acids in the available transcriptome reads (supplementary table S6, Supplementary Material online). *S. rosetta* appears to lack a ${\text{tRNA}}_{\text{AGT}}^{\text{Thr}}$ gene, therefore only tRNA molecules for leucine, isoleucine, valine, serine, proline, alanine, and arginine were screened for in this species. The number of tRNA transcripts identified in the transcriptomes was low, however across the two species tRNA molecules with guanine at the wobble position were identified for isoleucine, serine, proline, alanine, and arginine (supplementary table S6, Supplementary Material online). tRNA genes with guanine at the wobble position for those amino acids are absent from the genomes, therefore, it can be concluded that the holozoan protists are modifying their tRNA molecules through adenosine deamination. If, as proposed here, adenosine in tRNA anticodons is deaminated to inosine for all high degeneracy amino acids, the major tRNA gene for each amino acid will be complementary to the most frequently used optimal codons in all cases with the exception of threonine in the two choanoflagellates and lysine in *M. brevicollis*.

One unexpected finding was the identification of a putative tRNA gene for the amino acid selenocysteine in *C. owczarzaki* (supplementary table S4, Supplementary Material online), which possesses the anticodon TCA that should complement UGA codons. Analysis by tRNAScan-SE indicated that the tRNA^SeC^ gene is not a pseudogene, however the screening of both choanoflagellate genomes failed to identify any tRNA^SeC^ genes.

### Codon Usage Bias Increases with Expression Level in *S. rosetta* and *C. owczarzaki*

**Fig. 4.**
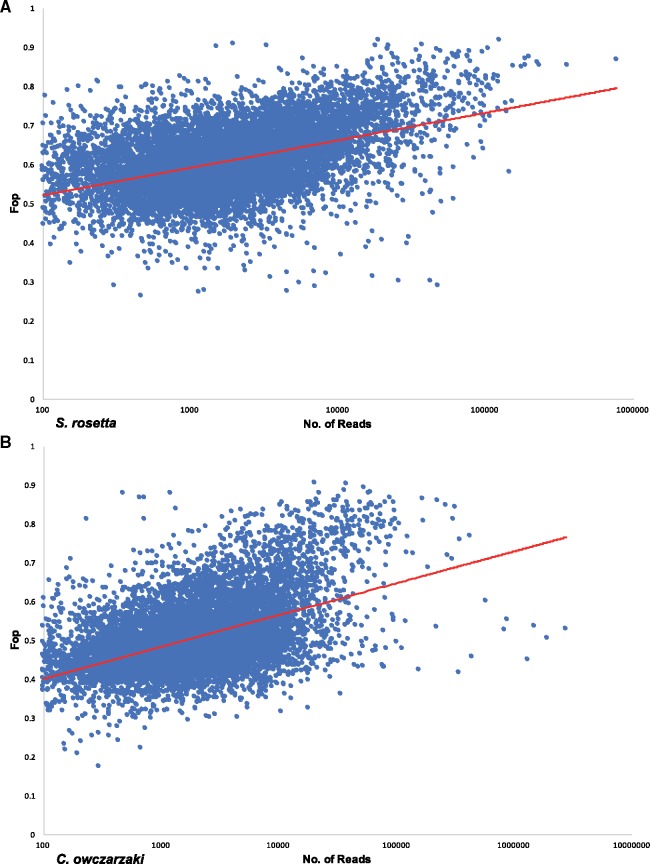
Relationships between gene expression levels and *F*_op_ in *S*. *rosetta* and *C. owczarzaki*. The *x*-axis (number of reads per gene) and line of best fit are both shown with a logarithmic scale for both species.

Deep coverage transcriptome data sets are available for both *S. rosetta* and *C. owczarzaki*, although not for *M. brevicollis*, and the number of reads for each gene was plotted against *F*_op_ for each gene. Figure 4 shows the relationships when genes with very low expression levels (<100 reads, which is similar to the level of pseudogene transcription observed in *C. owczarzaki* by Carr and Suga [2014]) have been excluded. In both species, there is a general trend observed for *F*_op_ to increase as the level of expression increases. The data therefore are consistent with selection operating upon translational efficiency, with highly expressed genes preferentially using optimal codons in comparison to weakly expressed genes. However, in both species *R*^2^ is low (0.218 for *S. rosetta* and 0.231 for *C. owczarzaki* after excluding genes with <100 reads), indicating that evolutionary forces in addition to selection for translational efficiency are operating in the genomes of *S. rosetta* and *C. owczarzaki*.

### Protein Domain Codons Exhibit Stronger Codon Usage Bias than Nondomain Codons

In order to determine if translational accuracy also plays a role, codon usage bias was examined in gene regions that encode structural or functional domains, as well as nondomain codons. Domains for each gene in the three bias categories were identified using the NCBI gene annotation and the degree of codon usage bias was determined using *F*_op_.

**Fig. 5.**
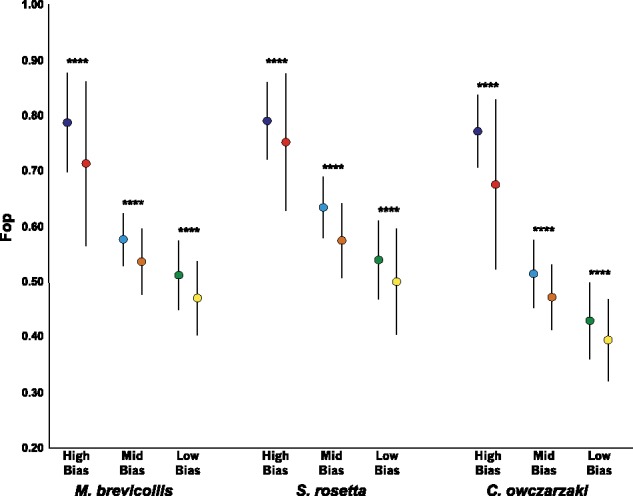
Mean *F*_op_ values in domain encoding and nondomain encoding codons. The dark blue dot gives the mean value for domain codons and the red dot shows the mean value for nondomain codons in highly biased genes. The light blue dot gives the mean value for domain codons and the orange dot shows the mean value for nondomain codons in mid-biased genes. The green dot gives the mean value for domain codons and the yellow dot shows the mean value for nondomain codons in low-biased genes. The bars represent the standard deviation of the values from each category. ****P <* *0.0001.

*F*_op_ was significantly elevated in domain codons compared with nondomain codons in all three categories (*t* test, *P *<* *0.0001 for each comparison) for all three species (fig. 5 and supplementary table S7, Supplementary Material online). The data therefore are consistent with selection for translational accuracy being a driver of codon usage in the three holozoan protists and that its effects are observable even in weakly biased genes.

### Estimates of the Strength of Translational Selection in the Three Holozoan Protists

All three genomes show evidence for selection acting upon translational accuracy and both *S. rosetta* and *C. owczarzaki*, for which expression data are available, show evidence for selection for translational efficiency. Numerous methodologies have been devised to determine the strength of selection acting upon codon usage and two are used here in an attempt to quantify selection within the holozoan protists. Eyre-Walker and Bulmer (1995) used the odds ratio for pairs of synonymous codons in high and low expressed genes (eq. 4 in that paper) to measure the strength of selection. This is adapted here to compare high and low bias genes, based upon Axis 1 of the correspondence analyses, since expression data are only available for two of the three species. Direct comparisons must be taken with caution, due to the different methodologies used to generate odd ratios, however the values produced for 2-fold degenerate amino acids (supplementary table S8, Supplementary Material online) are similar for the holozoan protists to those for enterobacteria determined by Eyre-Walker and Bulmer.

Sharp et al. (2005) developed a population genetics based model that results in the statistic *S*, which estimates the strength of selected codon usage bias. This was used to study selection in a broad range of bacterial taxa, whereas dos Reis and Wernisch (2009) noted that *S* is the log of Eyre-Walker and Bulmer’s odd ratio when estimating the strength of selection on codon usage in eukaryotes. This calculation is applied here to estimate *S* for each 2-fold amino acid, as well as for the genome using the weighted average for each 2-fold degenerate amino acid in the highly biased genes (table 1 and supplementary table S8, Supplementary Material online). The genome averages (*Ŝ*) for *S. rosetta* and *C. owczarzaki* are similar, if a little lower, to the estimates of dos Reis and Wernisch (2009) for Dikarya fungi. *M. brevicollis* exhibits a lower value for *Ŝ*, however it is comparable to values from ecdysozoans (dos Reis and Wernisch (2009), which have previously been shown to have selection upon their codon usage (reviewed in Duret 2000).

## Discussion

### Codon Usage Is Highly Conserved across the Three Holozoan Protists

Codon usage has been extensively studied in a broad range of opisthokont taxa, mainly centring on metazoans and fungi. The sequencing of whole genomes from unicellular holozoans provides an insight into the evolutionary forces that drive codon usage in the closest relatives of Metazoa. Molecular clock estimates suggest that choanoflagellates last shared a common ancestor with *C. owczarzaki* over 1 billion years ago (Parfrey et al. 2011). Despite this antiquity, many of the aspects of codon usage across the three holozoan protists examined are remarkably similar.

All three protists studied show a similar bias towards GC-ending codons and optimal codons which mainly end in either cytosine or guanine. Within 4-fold and 6-fold-degenerate amino acids, there is a clear preference for cytosine at the synonymous third position of codons. Every amino acid shares at least one optimal codon across the three species and only seven amino acids show any variation in their full complement of optimal codons (table 2). This suggests that codon usage is either highly conserved, or that there has been an extraordinary level of convergent evolution between the species.

One area in which *C. owczarzaki* differs from the two choanoflagellates is the presence of a tRNA gene for the amino acid selenocysteine (supplementary table S4, Supplementary Material online). The presence in the *C. owczarzaki* genome of tRNA^SeC^, which possesses an anticodon that complements UGA stop codons, suggests that the current annotation of selenoproteins may require revision; selenocysteine codons may have been identified as stop codons, resulting in erroneously truncated conceptual proteins. The 3′ untranslated regions of selenoprotein genes contain a Sec incorporation sequence (SECIS), which is required for the correct incorporation of selenocysteine in place of translation termination (reviewed in Shetty et al. 2014). SECIS screening the putative selenoprotein genes in the *C. owczarzaki* genome could highlight any proteins which have been misannotated with premature stop codons.

A second potential difference between species is the approximately 200 genes in the *M. brevicollis* genome which do not conform to the general trend of increasing GC3s with increasing bias (fig. 2 and supplementary fig. S1, Supplementary Material online). Further investigation of the genes, in particular undertaking reciprocal BLAST analyses, indicated that many of the genes may actually be artefacts generated during genome annotation. The use of *Nc* plots has the potential to aid genome annotation, particularly in species which exhibit strong trends in codon usage bias as observed here. Genes which are candidates for being false positives in genome annotation can be scrutinized in greater depth in order to determine if they are genuine. Furthermore, *Nc* plots may also have a role in identifying genes that are present due to horizontal transfer. Choanoflagellates are known to have acquired a large number of genes from algae and bacteria (Tucker 2013), possibly through the escape of prey DNA from food vacuoles into their nuclei. In cases where donor species have distinct codon usage from the recipient species, horizontally transferred genes are likely to be placed away from vertically inherited genes on *Nc* plots. This methodology however would be limited in only identifying recent transfers, as successfully transferred genes are likely to adapt their codon usage to that of their new host.

As is set out below, the forces that determine codon usage within the three holozoan protists appear to be extremely similar. Since the last common ancestor of choanoflagellates and filastereans was also a direct ancestor of Metazoa, the analyses presented here show how codon usage was likely to have been driven in unicellular premetazoans.

### Selection for Optimal Codons Is a Major Driver of Codon Usage Bias

There was a significant reduction in GC3s when comparing mid-bias to high-bias genes and low-bias to mid-bias genes, consistent with the enriched use of translationally optimal, GC-ending codons in highly biased genes. The noncoding GC content of the genes did not show the same pattern of significant reductions across gene bias categories, indicating that mutation pressure is not a major driver of codon usage bias in the genes of the three species.

The stop codons employed by highly biased genes do not show evidence of GC mutation pressure. Although the sense codons of highly biased genes show a strong for preference for GC at synonymous sites, the preferred stop codon of all three species is UAA. Brown et al (1990) noted that highly expressed genes in a broad variety of eukaryotes prefer UAA as a stop codon, however they also noted that species with GC-rich genomes had a preference for UGA as a stop codon. The only exception to GC-rich species preferring UGA was the unicellular *Chlamydomonas reinhardtii*, which exhibits a similar pattern to the three holzoan protists here in having a GC-rich genome and a preference for UAA stop codons in highly expressed genes. Brown et al. (1990) proposed a possible selective advantage in using UAA as a stop codon, since UGA may be misread as a UGG tryptophan codon. Furthermore, UGA and UAG are more likely than UAA to act as suppressible stop codons (Brown et al. 1990). Similar selective forces may be in operation within the GC-biased holozoan protist genomes, providing an explanation for the excess of UAA stop codons in highly biased genes.

Gene expression data from *S. rosetta* and *C. owczarzaki* were consistent with selection for translational efficiency in both species. Expression data are currently unavailable for *M. brevicollis*, but the KOG category that includes highly expressed protein translation genes is significantly enriched in high bias genes within this species (supplementary table S1, Supplementary Material online). Furthermore, across all three species, the major tRNA genes appear to be complementary, albeit after tRNA modification, with optimal codons. This also points to selection for translational efficiency, as it appears likely that the most abundant tRNA molecules in each species will be available to bind to optimal codons. The codon usage of highly biased genes and the number of tRNA genes therefore appear to have coevolved for the rapid synthesis of proteins within holozoan protists.

Genes with low expression levels show lower levels of enrichment for optimal codons, consistent with the predictions of selection for translational efficiency. However, even genes that show weak codon usage bias exhibit the signature of selection for translational accuracy, due to regions encoding functional domains being enriched for optimal codons, which is in agreement with recent findings in weakly expressed *E. coli* genes (Yannai et al. 2018). Selection for translational accuracy therefore also appears to be a driver of codon usage bias in choanoflagellates and filastereans.

Despite the different life histories of the free-living choanoflagellates and the symbiotic *C. owczarzaki*, all three holozoans exhibit similar patterns within their codon usage bias. If *C. owczarzaki* does undergo population bottlenecks when it passes from one host to another, they are not sufficiently severe to significantly dampen selection for optimal codons. Indeed, the estimated strength of selected codon usage bias (*Ŝ*), based upon 2-fold degenerate amino acids, is higher for *C. owczarzaki* than for either choanoflagellate. A further potential source of difference between the choanoflagellates and *C. owczarzaki* is the use of EFL as an elongation factor in the former, whereas the latter employs EF1A (Carr et al. 2017). The elongation factors facilitate the delivery of aminoacyl-tRNA molecules to the ribosome (Riis et al. 1990) and it could be speculated that the two proteins may exhibit differences in how they interact with tRNA molecules. However, as with the differences in life histories, the alternative elongation factors do not appear to have a noticeable impact on the tRNA genes or optimal codons of the holozoan protists.

Within the two choanoflagellates codon usage bias is stronger in *S. rosetta* than *M. brevicollis*. The presence of possible inaccurately annotated genes in *M. brevicollis* may however be acting to lower the apparent strength of codon usage bias within this species. If selection is genuinely weaker in *M. brevicollis*, the stronger bias in *S. rosetta* indicates that it may have a larger effective, if not absolute, population size. There is currently a lack of empirical evidence to test this hypothesis, with no ecological or population nucleotide diversity data available for either choanoflagellate species. Recent studies have shown that *S. rosetta* undergoes sexual reproduction (Levin and King 2013; Woznica et al. 2017), but, whereas there is indirect evidence of sexual reproduction in both *M. brevicollis* and *C. owczarzaki* (Carr et al. 2010; Suga et al. 2013), it has yet to be confirmed in either species. Differences in recombination rates, as a result of differences in the frequency of sexual reproduction, may influence the effective population sizes of the three holozoan protists, and therefore, the efficiency of selection upon optimal codons. The rates of cell division between the two species of choanoflagellate are also currently unknown and this component of life history may have an impact upon the strength of selection of translational efficiency, since rapid cell division is likely to require rapid protein synthesis. *M. brevicollis* is a strictly unicellular choanoflagellate (reviewed in Carr et al. 2017) and cellular differentiation has to be reported in this species; in contrast *S. rosetta* can form ephemeral colonies and produce at least five different cell types (Dayel et al. 2011). Whether the different *S. rosetta* cell types exhibit similar rates of cell division, how the rates compare to those of *M. brevicollis* and whether this influences selection on codon usage highlights the lack of current knowledge on much of choanoflagellate biology.

### Evidence for Widespread Deamination of Adenosine at the Wobble Position of tRNA Molecules

The major tRNA genes of 2-fold degenerate amino acids show a perfect complementary match to their optimal codons with the exception of lysine in *M. brevicollis*. Analysis of the *M. brevicollis* genome with tRNA-Scan-SE identified two tRNA^Lys^ genes, with one gene complementing each lysine codon (supplementary table S4, Supplementary Material online). In addition, the screen of the genome also identified five putative tRNA^Lys^ pseudogenes, all of which had CUU anticodons. The lack of a match between optimal codon and major tRNA for lysine may therefore have only arisen recently with the loss of function in the ${\text{tRNA}}_{\text{CUU}}^{\text{Lys}}$ pseudogenes.

Of the amino acids with higher levels of degeneracy in their genetic codes, tRNA genes with adenosine at the wobble position make up almost all of the major tRNA genes (table 2 and supplementary S3, Supplementary Material online). The most frequently used optimal codons for these amino acids show cytosine at the degenerate position, with the exception of UCG, which is the most frequently used optimal codon for serine in *C. owczarzaki*. It is clear that these optimal codons do not rely upon standard Watson–Crick base pairings to bind tRNA, since tRNA genes with guanine at the wobble position of anticodons are absent (supplementary table S5, Supplementary Material online). Complementation of codons and anticodons could be achieved through tRNA modification, with adenosine at the wobble position of the tRNA being converted to inosine. Rafels-Ybern et al. (2017) recently showed that the deamination of this adenosine is widespread amongst eukaryotes and that codons complementary to deaminated tRNA molecules are enriched in highly expressed genes. Glycine is the only amino acid where the phenomenon does not occur in eukaryotes, due to a lack of stability in the structure of ${\text{tRNA}}_{\text{ACC}}^{\text{Gly}}$ molecules (Yokoyama and Nishimura 1995; Saint-Léger et al. 2016). Glycine is also the only higher degeneracy amino acid which shows standard Watson–Crick base pairing between major tRNA gene and the most frequent optimal codon in the studied genomes (table 2). Transcriptome sequences contain cDNA sequences with guanine at the wobble position of anticodons. It therefore appears that adenosine modification is the most plausible explanation for the presence of these tRNA molecules in the transcriptomes. The transcriptome sequences also show the presence of tRNA molecules with adenine at the wobble position of anticodons, which have escaped deamination (supplementary table S6, Supplementary Material online). Those tRNAs will bind to uracil-ending codons under standard Watson–Crick base pairing. This is consistent with the occurrence of lower frequency, uracil-ending optimal codons for some higher degeneracy amino acids (supplementary table S3, Supplementary Material online) and provides a selection-based explanation for uracil-ending optimal codons.

The wide-scale deamination of adenosine to inosine is common across different RNA types in Metazoa (Porath et al. 2017) and Rafels-Ybern et al. (2017) showed that metazoan and plant genomes possess a greater concentration of codons translated by modified tRNAs than the genomes of unicellular eukaryotes. From this observation, they speculated that such codons were important in the evolution of multicellularity in metazoans and plants. However, based upon the analyses presented here, it appears that the large-scale usage of deaminated, tRNAs molecules evolved early within Holozoa prior to the emergence of true multicellularity.

### Optimizing Future Transgene Design within Holozoan Protists

The three holozoan protists studied here are lab workhorses in the study of the evolution of Holozoa and the origin of metazoan multicellularity (Abedin and King 2008; Carr et al. 2010; Najile et al. 2016; Sebé-Pedrós et al. 2016; Woznica et al. 2016). Transgenics systems are currently being developed for holozoan protists (Parra-Acero et al. 2018), and the data presented here provide important information for the expression of artificial transgenes. The use of a species’ optimal codons in a transgene can increase the yield of the encoded protein by three orders of magnitude (Gustafsson et al. 2012) and table 2 presents the optimal codons, as well as preferred stop codons, for all three species, enabling the future tailored design of transgenes for each of the holozoans.

## Conclusions

The genome analyses presented highlight the role of natural selection in the evolution of codon usage bias within extant holozoan protists. Importantly, the data also contribute to the growing knowledge of unicellular, premetazoan evolution. The data indicate that natural selection operating upon both translational accuracy and efficiency resulted in a GC-bias in the codon usage bias of premetazoans. It is increasingly clear that the modification of RNA molecules through adenosine deamination to inosine is important in extant metazoans. However, it can be seen that tRNA molecules for higher level degeneracy amino acids underwent deamination in the very earliest premetazoans and that this trait evolved prior to the divergence of Filasterea from the lineage that lead to both choanoflagellates and metazoans. Furthermore, highly biased genes in early metazoan evolutionary history would have been enriched with codons that complemented the modified tRNA molecules.

The transition from unicellular premetazoan to multicellular stem-group metazoan is likely to have resulted in a number of changes that led to a reduction of efficient selection for optimal codons. In particular, the effective population sizes of metazoans are believed to be lower than those of unicellular species, resulting in less efficient selection. Longer generation times, due to the division of somatic and germ line cells and the development of adult life-stages, are also likely to have reduced selection for rapid cell division, thereby relaxing selective pressures for translational efficiency. Finally, a differentiated multicellular bodyplan allowed the evolution of tissue-specific tRNA expression patterns, breaking the link between tRNA gene number and expression level.

## Materials and Methods

All analyzed data sets are available from the corresponding author upon request.

### Codon Usage Statistics

Complete annotated transcriptome sequences for *M. brevicollis* and *S. rosetta* were downloaded from the Origins of Multicellularity Project at the Broad Institute; the *C. owczarzaki* transcriptome was downloaded from the EnsemblProtists database. The versions used were: monosiga_brevicollis_mx1_1_transcripts, salpingoeca_rosetta_1_transcripts and Capsaspora_owczarzaki_atcc_30864.C_owczarzaki_V2.cds. Codon usage statistics were determined in CodonW (Peden 1999). Optimal codon, fop.coa files, were generated by correspondence analyses, using RSCU, of the complete transcriptomes of each species using default parameters. *S. rosetta* and *C. owczarzaki* both have large publicly available data sets of gene expression (see Gene Expression in *S. rosetta* and *C. owczarzaki* below) and in both species the principal axis of the correspondence analysis shows the strongest relationship with expression, consistent with selection operating upon translational efficiency (Peden, 1999). Optimal codons were also determined using expression levels (Lloyd and Sharp 1991, 1993; Stenico et al. 1994), by identifying codons present at significantly higher frequencies in the 5% most highly expressed genes compared with the 5% most weakly expressed genes. Values of *Nc*, GC3s and *F*_op_ were calculated in CodonW. *F*_op_ values were determined using the fop.coa file produced by CodonW for *M. brevicollis* and the optimal codons determined using expression levels for *S. rosetta* and *C. owczarzaki*.

Odds ratios between synonymous codon pairs within the 5% most biased genes and the least biased genes, based upon the primary axes of the CodonW correspondence analyses were determined for all 2-fold degenerate amino acids. The population parameter for the strength of codon usage bias, *S*, was estimated for each of the nine 2-fold degenerate amino acid by determining the log of the odds ratio values. Species estimates, *Ŝ*, were calculated by determining the weighted average for amino acids based upon the number of codons in highly biased genes (based upon Sharp et al. 2005).

### Determining Non-Coding GC Content

The genes in each bias-category were also screened for predicted introns in the NCBI gene annotations. When possible 200 bp of both 5′ and 3′ flanking DNA was extracted for each in the three bias categories. Where predicted intergenic regions were <200 bp, or genes were located at the ends of genomic contigs, the maximum possible length of flanking DNA was extracted. All introns and flanking DNA for each gene were extracted, concatenated, and their total GC content was calculated in CodonW. Mean and standard deviation values were then generated for each category.

### tRNA Gene Screening

The scaffolds of each annotated genome were downloaded from NCBI. The *M. brevicollis* data set was made up from the 218 scaffolds of the version 1 genome assembly, *S*. *rosetta* data set comprised the 3,086 scaffolds of the version 1 assembly and the *C. owczarzaki* data set was the 84 scaffolds from the version 2 assembly. The program tRNAscan-SE 2.0 (Lowe and Eddy, 1997), applied through an online server (Lowe and Chan 2016), was used to identify tRNA genes using default settings.

### Gene Expression in *S. rosetta* and *C. owczarzaki*

The level of gene expression for each species was determined by the examining the high, medium and low bias categories, using SMALT v. 0.2.6 (Hannes Ponstingl, Genome Research Ltd) in both *S. rosetta* and *C. owczarzaki*. For *S. rosetta* the transcriptomic SRA files SRX042046-SRX042054 (122.1 million reads) and for *C. owczarzaki* SRX1690425-SRX1690428 and SRX155789-SRX155797 (665.2 million reads) were downloaded from the NCBI SRA database. SRA reads were mapped onto each gene sequence to determine expression level. The number of reads for each gene was then calculated in Tablet v. 1.16.09.06 (Milne et al. 2013). Reads were additionally mapped on to the tRNA genes of alanine, arginine, isoleucine, leucine, proline, serine, threonine, and valine that possess adenosine and guanosine at the wobble position.

### Optimal Codon Usage in Domain and Nondomain Codons

Within the bias-categories, each gene was divided into putative functional domain and nondomain codons. Codons encoding functional domains were identified from the annotated Regions within the NCBI file for each gene. Genes which did not encode annotated regions were excluded from the analyses; furthermore, genes were excluded when the defined region spanned the entire gene, or all codons with the exception of the start and/or the stop codon. *F*_op_ values were determined in CodonW for the functional domain and nondomain regions. Values of *Nc* were not considered, as some regions did not contain codons from all of the required redundancy categories.

## Supplementary Material

Supplementary Data
