# Supplementary material for: Patterns of Ancestral Animal Codon Usage Bias Revealed through Holozoan Protists
Source: Mol Biol Evol. 2018 Aug 29;35(10):2499–511. doi: 10.1093/molbev/msy157 (PMC6188563; doi:10.1093/molbev/msy157)
Supplement: Supplementary Data [file msy157_supp.zip › Southworth_et_al_SM.pdf]

Supplementary Information

Patterns of Ancestral Animal Codon Usage Bias Revealed Through Holozoan Protists

Figure S1-S2 Legends

Supplementary Tables S1-S8

Supplementary Figure Legends

Figure S1. ***M. brevicollis Nc*** **Plot.** Genes which fall outside the main cluster were investigated with reciprocal BLASTs. Red dots denote genes which were not recovered with reciprocal BLAST, green dots show genes which were recovered. Blue dots denote genes which were not examined.

Figure S2. Frequency spectra of GC content in putatively neutral sites. The first nine graphs show the GC content spectra for synonymous codon third positions, graphs 10 to 18 show intron data and graphs 19-27 show flanking DNA data.

Supplementary Tables

Table S1. Gene ontology (KOG) percentage breakdown in the three bias categories.

KOG Category	High Bias	Mid Bias	Low Bias	*P* Values
**1) Cellular Processes And Signaling**	**28.18**	**33.71**	**35.60**	**NS, NS**
M: Cell wall/membrane/envelope biogenesis	2.76	2.29	2.26	NS, NS
N: Cell motility	0.00	0.29	0.00	NS, NS
O: Posttranslational modification, protein turnover, chaperones	10.50	5.14	9.47	<0.01, NS
T: Signal transduction mechanisms	5.52	14.29	12.50	<0.0001, <0.005
U: Intracellular trafficking, secretion, and vesicular transport	1.93	3.14	2.65	NS, NS
V: Defense mechanisms	0.83	1.71	1.14	NS, NS
W: Extracellular structures	1.66	1.43	2.27	NS, NS
Y: Nuclear structure	0.00	1.14	0.00	NS, NS
Z: Cytoskeleton	4.97	4.29	5.30	NS, NS
**2) Information Storage And Processing**	**34.25**	**16.86**	**25.00**	**<0.0001, <0.02**
A: RNA processing and modification	4.97	4.86	6.44	NS, NS
B: Chromatin structure and dynamics	4.14	0.03	4.55	0.0005, NS
J: Translation, ribosomal structure and biogenesis	18.23	4.57	3.03	<0.0001, <0.0001
K: Transcription	6.63	2.57	7.58	<0.02, NS

L: Replication, recombination and repair	0.28	4.57	3.48	<0.0001, <0.005
**3) Metabolism**	**31.77**	**22.00**	**11.74**	**<0.005, <0.0001**
C: Energy production and conversion	9.67	1.42	4.55	<0.0001, <0.0001
D: Cell cycle control, cell division, chromosome partitioning	4.70	4.00	1.14	NS, NS
E: Amino acid transport and metabolism	6.36	2.57	0.38	<0.02, <0.0001
F: Nucleotide transport and metabolism	1.66	0.86	0.38	NS, NS
G: Carbohydrate transport and metabolism	1.66	5.14	2.27	<0.02, NS
H: Coenzyme transport and metabolism	0.55	1.14	0.76	NS, NS
I: Lipid transport and metabolism	5.25	3.71	0.76	NS, <0.002
P: Inorganic ion transport and metabolism	1.10	2.57	1.89	NS, NS
Q: Secondary metabolites biosynthesis, transport and catabolism	0.83	0.57	0.38	NS, NS
**4) Poorly Characterized**	**5.80**	**27.43**	**27.65**	**<0.0001, <0.0001**
R: General function prediction only	4.14	16.00	18.18	<0.0001, <0.0001
S: Function unknown	1.66	11.43	9.47	<0.0001, 0.0005

Note: The *P* value are generated from Fisher exact tests comparing the frequency of categories in high bias genes to mid (left) and low (right) bias genes. Green text highlights categories which are significantly enriched in highly biased genes. Red font denotes categories which are significantly underrepresented in highly biased genes.

Table S2. GC3s, exonic and intronic GC content values from the three bias categories in *M. brevicollis*, *S. rosetta* and *C. owczarzaki*.

Species	GC3s (±sd)	Flanking GC Content (±sd)	Intron GC Content (±sd)
M. brevicollis			
High Bias	0.724±0.109	0.525±0.043	0.536±0.048
Mid Bias	0.635±0.031	0.527±0.040	0.543±0.031
Low Bias	0.580±0.043	0.520±0.048	0.531±0.040
High vs Mid	n/a	0.653	0.047
Mid vs Low	<0.0001	0.025	<0.0001
S. rosetta			
High Bias	0.829±0.056	0.535±0.030	0.537±0.036
Mid Bias	0.701±0.043	0.524±0.031	0.531±0.023
Low Bias	0.609±0.054	0.523±0.033	0.529±0.026
High vs Mid	<0.0001	<0.0001	0.005
Mid vs Low	<0.0001	0.909	0.449
C. owczarzaki			
High Bias	0.804±0.051	0.436±0.042	0.471±0.055
Mid Bias	0.643±0.038	0.447±0.048	0.478±0.041
Low Bias	0.555±0.051	0.464±0.047	0.488±0.042
High vs Mid	<0.0001	<0.0001	0.024
Mid vs Low	<0.0001	<0.0001	0.0005

Note: The *P* values from *t* tests on comparisons of high and mid bias genes, as well as mid and low bias genes are shown below the GC content values for each species.

Table S3. Output of optimal codon analyses from CodonW in each holozoan protist.

S3a. *M. brevicollis* Codon Usage

		**Highly Biased Genes**		**Weakly Biased Genes**				**Highly Biased Genes**		**Weakly Biased Genes**	
**Amino Acid**	**Codon Sequence**	**No. of Codons**	**RSCU**	**No. of Codons**	**RSCU**	**Amino Acid**	**Codon Sequence**	**No. of Codons**	**RSCU**	**No. of Codons**	**RSCU**
**Phe**	UUU	3077	0.91	2505	1.22	**Tyr**	UAU	863	0.34	1180	0.88
	UUC	3682	1.09	1615	0.78		UAC	4160	1.66	1498	1.12
**Leu**	UUA	12	0.01	600	0.21	**Stop**	UAA	352	2.43	128	0.58
	UUG	1258	0.56	2890	1.03		UAG	49	0.34	101	0.46
	CUU	2058	0.91	2425	0.86	**His**	CAU	761	0.43	1984	0.88
	CUC	5890	2.62	5579	1.98		CAC	2809	1.57	2506	1.12
	CUA	94	0.04	1487	0.53	**Gln**	CAA	1427	0.46	3501	0.97
	CUG	4187	1.86	3931	1.39		CAG	4728	1.54	3689	1.03
**Ile**	AUU	3417	1.23	1654	1.14	**Asn**	AAU	672	0.20	1747	0.82
	AUC	4892	1.76	2112	1.45		AAC	6035	1.80	2523	1.18
	AUA	10	0.00	604	0.41	**Lys**	AAA	443	0.09	2241	0.80
Met	AUG	4264	1.00	2808	1.00		AAG	9003	1.91	3352	1.20
**Val**	GUU	2353	0.79	1754	0.74	**Asp**	GAU	4025	0.77	3480	0.95
	GUC	6120	2.04	2390	1.01		GAC	6490	1.23	3874	1.05
	GUA	122	0.04	990	0.42	**Glu**	GAA	1869	0.36	3018	0.67
	GUG	3387	1.13	4340	1.83		GAG	8588	1.64	5935	1.33
**Ser**	UCU	1209	0.72	4956	2.08	**Cys**	UGU	314	0.23	2341	1.15
	UCC	3019	1.79	1484	0.62		UGC	2411	1.77	1746	0.85
	UCA	522	0.31	1944	0.81	**Stop**	UGA	34	0.23	434	1.96
	UCG	2412	1.43	1783	0.75	**Trp**	UGG	1849	1.00	1680	1.00

**Pro**	CCU	1398	0.73	1795	0.89	**Arg**	CGU	2586	1.77	1590	0.89
	CCC	5256	2.75	2163	1.07		CGC	5824	3.98	2400	1.35
	CCA	305	0.16	2469	1.22		CGA	171	0.12	1977	1.11
	CCG	690	0.36	1674	0.83		CGG	162	0.11	1565	0.88
**Thr**	ACU	1662	0.67	1448	0.63	**Ser**	AGU	280	0.17	1381	0.58
	ACC	6340	2.55	2787	1.21		AGC	2684	1.59	2773	1.16
	ACA	348	0.14	2686	1.16	**Arg**	AGA	8	0.01	1998	1.12
	ACG	1599	0.64	2318	1.00		AGG	37	0.03	1143	0.64
**Ala**	GCU	3766	0.94	3338	0.96	**Gly**	GGU	5027	1.56	1813	0.86
	GCC	10166	2.54	4212	1.21		GGC	7404	2.29	3218	1.53
	GCA	630	0.16	3487	1.01		GGA	346	0.11	1729	0.82
	GCG	1443	0.36	2837	0.82		GGG	150	0.05	1666	0.79

S3b. *S. rosetta* Codon Usage

		**Highly Biased Genes**		**Weakly Biased Genes**				**Highly Biased Genes**		**Weakly Biased Genes**	
**Amino Acid**	**Codon Sequence**	**No. of Codons**	**RSCU**	**No. of Codons**	**RSCU**	**Amino Acid**	**Codon Sequence**	**No. of Codons**	**RSCU**	**No. of Codons**	**RSCU**
**Phe**	UUU	4458	0.74	2471	1.05	**Tyr**	UAU	763	0.18	1110	0.77
	UUC	7544	1.26	2217	0.95		UAC	7926	1.82	1787	1.23
**Leu**	UUA	11	0.00	464	0.18	**Stop**	UAA	298	1.53	104	0.54
	UUG	620	0.15	3577	1.39		UAG	65	0.33	132	0.68
	CUU	3068	0.75	2379	0.92	**His**	CAU	552	0.16	2717	0.64
	CUC	11256	2.76	3195	1.24		CAC	6173	1.84	5783	1.36
	CUA	79	0.02	689	0.27	**Gln**	CAA	866	0.15	8128	0.95
	CUG	9435	2.31	5139	2.00		CAG	10900	1.85	8911	1.05
**Ile**	AUU	4498	0.99	1804	1.11	**Asn**	AAU	366	0.06	1540	0.57
	AUC	9111	2.00	2433	1.50		AAC	11636	1.94	3870	1.43
	AUA	26	0.01	623	0.38	**Lys**	AAA	383	0.05	2046	0.55
Met	AUG	7063	1.00	4303	1.00		AAG	15065	1.95	5384	1.45
**Val**	GUU	3505	0.60	2589	0.83	**Asp**	GAU	3221	0.33	6224	0.95
	GUC	8926	1.53	2560	0.82		GAC	16429	1.67	6935	1.05
	GUA	118	0.02	957	0.31	**Glu**	GAA	1197	0.13	4333	0.75
	GUG	10721	1.84	6316	2.03		GAG	17626	1.87	7229	1.25
**Ser**	UCU	2344	0.69	1811	0.62	**Cys**	UGU	419	0.15	1713	0.87
	UCC	7112	2.10	2311	0.79		UGC	5283	1.85	2222	1.13
	UCA	637	0.19	3594	1.23	**Stop**	UGA	220	1.13	344	1.78
	UCG	3792	1.12	2991	1.03	Trp	UGG	3296	1.00	2049	1.00
**Pro**	CCU	1666	0.44	2056	0.67	**Arg**	CGU	1749	0.64	2360	0.90
	CCC	6902	1.83	2246	0.74		CGC	12528	4.60	4154	1.58

	CCA	2256	0.60	5052	1.66		CGA	233	0.09	2842	1.08
	CCG	4227	1.12	2840	0.93		CGG	1232	0.45	2622	1.00
**Thr**	ACU	1234	0.23	1584	0.42	**Ser**	AGU	350	0.10	2013	0.69
	ACC	10340	1.90	3235	0.86		AGC	6106	1.80	4784	1.64
	ACA	2273	0.42	5934	1.58	**Arg**	AGA	80	0.03	1677	0.64
	ACG	7919	1.46	4254	1.13		AGG	527	0.19	2106	0.80
**Ala**	GCU	3445	0.48	3654	0.67	**Gly**	GGU	3152	0.59	4400	1.19
	GCC	15942	2.21	4418	0.81		GGC	16179	3.03	5080	1.37
	GCA	3115	0.43	8119	1.49		GGA	613	0.11	2596	0.70
	GCG	6347	0.88	5665	1.04		GGG	1400	0.26	2740	0.74

S3c. *C. owczarzaki* Codon Usage

Amino Acid	Codon Sequence	Highly Biased Genes No. of Codons	Highly Biased Genes RSCU	Weakly Biased Genes No. of Codons	Weakly Biased Genes RSCU	Amino Acid	Codon Sequence	Highly Biased Genes No. of Codons	Highly Biased Genes RSCU	Weakly Biased Genes No. of Codons	Weakly Biased Genes RSCU
**Phe**	**UUU**	2792	0.68	2731	1.13	**Tyr**	UAU	579	0.20	1335	0.97
	**UUC**	5402	1.32	2093	0.87		UAC	5251	1.80	1409	1.03
**Leu**	**UUA**	3	0.00	715	0.28	**Stop**	UAA	442	2.65	125	0.76
	**UUG**	894	0.31	3381	1.32		UAG	20	0.12	107	0.65
	**CUU**	2415	0.84	2640	1.03	**His**	CAU	313	0.14	1843	0.96
	**CUC**	9922	3.44	3606	1.41		CAC	4091	1.86	2003	1.04
	**CUA**	20	0.01	997	0.39	**Gln**	CAA	1608	0.42	4063	0.98
	**CUG**	4065	1.41	4024	1.57		CAG	5982	1.58	4235	1.02
**Ile**	**AUU**	4439	1.26	3604	1.66	**Asn**	AAU	293	0.07	3098	0.97
	**AUC**	6156	1.74	3606	1.07		AAC	8205	1.93	3288	1.03
	**AUA**	8	0.00	997	0.27	**Lys**	AAA	223	0.03	2750	0.99
Met	**AUG**	4053	1.00	9932	1.00		AAG	12743	1.97	2814	1.01
**Val**	**GUU**	3024	0.83	2997	1.13	**Asp**	GAU	2161	0.38	4089	1.00
	**GUC**	9563	2.61	2853	1.08		GAC	9177	1.62	4051	1.00
	**GUA**	25	0.01	957	0.36	**Glu**	GAA	1638	0.27	3409	0.88
	**GUG**	2017	0.55	3758	1.42		GAG	10371	1.73	4335	1.12
**Ser**	**UCU**	2015	0.92	1915	0.80	**Cys**	UGU	59	0.05	1197	0.84
	**UCC**	3533	1.60	2219	0.93		UGC	2442	1.95	1167	1.16
	**UCA**	231	0.10	2265	0.95	**Stop**	UGA	39	0.23	259	1.58
	**UCG**	4847	2.20	3020	1.26	Trp	UGG	2084	1.00	1769	1.00
**Pro**	**CCU**	1062	0.48	1919	0.91	**Arg**	CGU	1954	1.14	1568	0.94
	**CCC**	5696	2.57	1520	0.72		CGC	7776	4.54	2183	1.31
	**CCA**	372	0.17	2826	1.34		CGA	396	0.23	2538	1.53
	**CCG**	1732	0.78	2190	1.04		CGG	81	0.05	1700	1.02
**Thr**	**ACU**	2668	0.90	2456	0.97	**Ser**	AGU	115	0.05	2047	0.86

	**ACC**	7231	2.44	2179	0.86		AGC	2470	1.12	2882	1.21
	**ACA**	288	0.10	2598	1.03	**Arg**	AGA	11	0.01	1040	0.63
	**ACG**	1673	0.56	2864	1.13		AGG	48	0.03	950	0.57
**Ala**	**GCU**	5992	1.05	4593	1.02	**Gly**	GGU	2636	0.76	1992	0.87
	**GCC**	13396	2.35	3549	0.79		GGC	10824	3.13	3199	1.40
	GCA	1158	0.20	4850	1.08		GGA	301	0.09	2411	1.06
	GCG	2236	0.39	4968	1.11		GGG	68	0.02	1536	0.67

Table S4. Output of tRNAscan-SE from whole genome contigs.

S4a. Predicted *Monosiga brevicollis* tRNA genes.

**Scaffold**	**Accession Number**	**tRNA Gene**	**Codon/tRNA Anti-codon**	**Codonw Optimal Codon**	**Co-ordinates**	**Putative Pseudogene**
MONBRscaffold_2	CH991543	Val	GUG/CAC	No	2094573-2094501	No
MONBRscaffold_2	CH991543	Val	GUG/CAC	No	2094768-2094696	No
MONBRscaffold_2	CH991543	Val	GUU/AAC	No	2095091-2095019	No
MONBRscaffold_2	CH991543	Thr	ACG/CGT	No	2422150-2422221	No
MONBRscaffold_3	CH991544	Leu	UUA/UAA	No	792492-792410	No
MONBRscaffold_3	CH991544	Arg	CGU/ACG	Yes	1049949 -1049876	No
MONBRscaffold_3	CH991544	Arg	CGU/ACG	Yes	1150184-1150111	No
MONBRscaffold_3	CH991544	Arg	CGU/ACG	Yes	1150507-1150434	No
MONBRscaffold_3	CH991544	Arg	CGU/ACG	Yes	1150707-1150634	No
MONBRscaffold_3	CH991544	Arg	CGU/ACG	Yes	1150891-1150818	No
MONBRscaffold_3	CH991544	Ile	AUU/AAT	No	2156667-2156594	No
MONBRscaffold_3	CH991544	Ile	AUU/AAT	No	2157029-2156956	No
MONBRscaffold_4	CH991545	Thr	ACG/CGT	No	111613-111542	No
MONBRscaffold_4	CH991545	Arg	CGA/UCG	No	315999-315927	No
MONBRscaffold_4	CH991545	His	CAC/GTG	Yes	1805888-1805958	No
MONBRscaffold_4	CH991545	His	CAC/GTG	Yes	1806150-1806220	No
MONBRscaffold_4	CH991545	His	CAC/GTG	Yes	1806545-1806615	No
MONBRscaffold_5	CH991546	Pro	CCU/AGG	No	695230-695159	No
MONBRscaffold_5	CH991546	Pro	CCU/AGG	No	695423-695352	No
MONBRscaffold_5	CH991546	Pro	CCU/AGG	No	695597-695526	No
MONBRscaffold_5	CH991546	Lys	AAG/CTT	Yes	857521-857442	No

MONBRscaffold_6	CH991547	Cys	UGC/GCA	Yes	935797-935718	No
MONBRscaffold_6	CH991547	Cys	UGC/GCA	Yes	936035-935967	No
MONBRscaffold_6	CH991547	Leu	UUG/CAA	No	964475-964395	No
MONBRscaffold_6	CH991547	Leu	UUG/CAA	No	964677-964597	No
MONBRscaffold_6	CH991547	Leu	CUG/CAG	Yes	986392-986472	No
MONBRscaffold_6	CH991547	Leu	CUG/CAG	Yes	986572-986652	No
MONBRscaffold_6	CH991547	Leu	CUG/CAG	Yes	986878-986958	No
MONBRscaffold_7	CH991548	Gly	GGC/GCC	Yes	431060-431130	No
MONBRscaffold_7	CH991548	Leu	CUA/TAG	No	726957-727037	No
MONBRscaffold_8	CH991549	Ala	GCG/CGC	No	411478-411549	No
MONBRscaffold_8	CH991549	Ala	GCG/CGC	No	411667-411738	No
MONBRscaffold_8	CH991549	Ala	GCA/TGC	No	411877-411948	No
MONBRscaffold_8	CH991549	Ala	GCA/TGC	No	412059-412130	No
MONBRscaffold_10	CH991551	Ser	AGC/GCT	Yes	212569-212642	No
MONBRscaffold_10	CH991551	Ser	AGC/GCT	Yes	212996-213069	No
MONBRscaffold_12	CH991553	Tyr	UAC/GTA	Yes	220550-220465	No
MONBRscaffold_12	CH991553	Arg	AGA/TCT	No	353701-353629	No
MONBRscaffold_13	CH991554	Glu	GAG/CTC	Yes	414502-414573	No
MONBRscaffold_13	CH991554	Glu	GAG/CTC	Yes	414673-414744	No
MONBRscaffold_13	CH991554	Glu	GAG/CTC	Yes	414847-414918	No
MONBRscaffold_13	CH991554	Glu	GAG/CTC	Yes	415537-415608	No
MONBRscaffold_13	CH991554	Arg	CGA/TCG	No	947577-947505	No
MONBRscaffold_14	CH991555	Ser	UCG/CGA	Yes	217567-217486	No
MONBRscaffold_14	CH991555	Ser	UCU/AGA	No	219199-219118	No
MONBRscaffold_14	CH991555	Ser	UCU/AGA	No	220213-220132	No
MONBRscaffold_14	CH991555	Ser	UCA/TGA	No	231571-231490	No

MONBRscaffold_14	CH991555	Ser	UCG/CGA	Yes	234840-234921	No
MONBRscaffold_14	CH991555	Ile	AUU/AAT	No	1036763-1036836	No
MONBRscaffold_15	CH991556	Glu	GAA/TTC	No	945958-946029	No
MONBRscaffold_15	CH991556	Glu	GAA/TTC	No	946151-946222	No
MONBRscaffold_16	CH991557	Met	AUG/CAT	n/a	423588-423659	No
MONBRscaffold_16	CH991557	Met	AUG/CAT	n/a	423951-424022	No
MONBRscaffold_16	CH991557	Ala	GCU/AGC	No	886446-886535	No
MONBRscaffold_16	CH991557	Ala	GCU/AGC	No	886655-886744	No
MONBRscaffold_16	CH991557	Ala	GCU/AGC	No	884519-894609	No
MONBRscaffold_17	CH991558	Gln	CAA/TTG	No	251303-251374	No
MONBRscaffold_17	CH991558	Gln	CAA/TTG	No	251503-251574	No
MONBRscaffold_17	CH991558	Glu	GAG/CTC	Yes	714134-714063	No
MONBRscaffold_18	CH991559	Leu	CUU/AAG	No	625719-625638	No
MONBRscaffold_18	CH991559	Leu	CUU/AAG	No	626521-626440	No
MONBRscaffold_18	CH991559	Leu	CUU/AAG	No	626928-626847	No
MONBRscaffold_18	CH991559	Leu	CUU/AAG	No	627265-627184	No
MONBRscaffold_21	CH991562	Ile	AUA/TAT	No	348454-348320	No
MONBRscaffold_21	CH991562	Ala	GCU/AGC	No	634940-635029	No
MONBRscaffold_21	CH991562	Tyr	UAC/GTA	Yes	683103-683188	No
MONBRscaffold_21	CH991562	Tyr	UAC/GTA	Yes	683494-683579	No
MONBRscaffold_22	CH991563	Val	GUA/TAC	No	106687-106615	No
MONBRscaffold_23	CH991564	Phe	UUC/GAA	Yes	536169-536241	No
MONBRscaffold_23	CH991564	Phe	UUC/GAA	Yes	552611-552683	No
MONBRscaffold_23	CH991564	Phe	UUC/GAA	Yes	553023-553095	No
MONBRscaffold_23	CH991564	Phe	UUC/GAA	Yes	553464-553536	No
MONBRscaffold_24	CH991565	Ser	UCU/AGA	No	340568-340649	No

MONBRscaffold_25	CH991566	Gly	GGA/TCC	No	334184-334076	Yes
MONBRscaffold_25	CH991566	Val	GUU/AAC	No	589695-589767	No
MONBRscaffold_25	CH991566	Val	GUU/AAC	No	600688-600760	No
MONBRscaffold_25	CH991566	Val	GUU/AAC	No	658300-658228	No
MONBRscaffold_26	CH991567	Lys	AAG/CTT	Yes	250951-251041	Yes
MONBRscaffold_26	CH991567	Lys	AAG/CTT	Yes	251372-251462	Yes
MONBRscaffold_26	CH991567	Lys	AAG/CTT	Yes	252098-252190	Yes
MONBRscaffold_26	CH991567	Lys	AAG/CTT	Yes	252651-252741	Yes
MONBRscaffold_26	CH991567	Lys	AAA/TTT	No	253229-253157	No
MONBRscaffold_29	CH991570	Glu	GAG/CTC	Yes	310919-310848	No
MONBRscaffold_29	CH991570	Arg	CGT/ACG	Yes	371239-371166	No
MONBRscaffold_30	CH991571	Met	ATG/CAT	n/a	542310-542238	No
MONBRscaffold_31	CH991572	Ala	GCT/AGC	No	52832-52922	No
MONBRscaffold_31	CH991572	Ala	GCT/AGC	No	53028-53117	No
MONBRscaffold_32	CH991573	Asp	GAC/GTC	Yes	352916-352845	No
MONBRscaffold_32	CH991573	Asp	GAC/GTC	Yes	369064-369135	No
MONBRscaffold_33	CH991574	Gln	CAG/CTG	Yes	143534-143463	No
MONBRscaffold_33	CH991574	Gln	CAG/CTG	Yes	144100-144029	No
MONBRscaffold_33	CH991574	Gln	CAG/CTG	Yes	204273-204344	No
MONBRscaffold_36	CH991577	Lys	AAG/CTT	Yes	54796-54631	Yes
MONBRscaffold_38	CH991579	Asp	GAC/GTC	Yes	259272-259343	No
MONBRscaffold_38	CH991579	Asp	GAC/GTC	Yes	259442-259513	No
MONBRscaffold_38	CH991579	Asp	GAC/GTC	Yes	259633-259704	No
MONBRscaffold_38	CH991579	Asp	GAC/GTC	Yes	259813-259884	No
MONBRscaffold_41	CH991582	Cys	UGC/GCA	Yes	44495-44567	
MONBRscaffold_42	CH991583	Gly	GGC/GCC	Yes	240173-240103	No

MONBRscaffold_42	CH991583	Gly	GGG/CCC	No	240351-240281	No
MONBRscaffold_42	CH991583	Gly	GGC/GCC	Yes	240546-240476	No
MONBRscaffold_42	CH991583	Gly	GGC/GCC	Yes	240866-240796	No
MONBRscaffold_42	CH991583	Gly	GGC/GCC	Yes	241087-241017	No
MONBRscaffold_43	CH991584	Pro	CCA/TGG	No	229064-229135	No
MONBRscaffold_52	CH991596	Gly	GGC/GCC	Yes	30699-30629	No
MONBRscaffold_52	CH991596	Gly	GGC/GCC	Yes	31029-30959	No
MONBRscaffold_55	CH991596	Pro	CCG/CGG	No	46181-46063	No
MONBRscaffold_55	CH991596	Trp	TGG/CCA	n/a	49232-49304	No
MONBRscaffold_55	CH991596	Trp	TGG/CCA	n/a	49484-49556	No
MONBRscaffold_49	CH991590	Asn	AAC/GTT	Yes	125396-125469	No
MONBRscaffold_49	CH991590	Asn	AAC/GTT	Yes	126199-126272	No
MONBRscaffold_49	CH991590	Asn	AAC/GTT	Yes	131693-131766	No

S4b. Predicted *S. rosetta* tRNA genes.

Scaffold	Accession Number	tRNA Gene	Codon/tRNA Anti-codon	Codonw Optimal Codon	Co-ordinates	Putative Pseudogene
cont1. 52	ACSY01000052	Ile	AUA/TAT	No	3073-2968	No
cont1. 83	ACSY01000083	Ile	AUU/AAT	No	120596-120669	No
cont1. 84	ACSY01000084	Ile	AUU/AAT	No	937-1010	No
cont1. 85	ACSY01000085	Ile	AUU/AAT	No	925-852	No
cont1. 86	ACSY01000086	Gln	CAA/TTG	No	14672-14743	No
cont1. 90	ACSY01000090	Leu	CUG/CAG	Yes	46873-46955	No
cont1. 90	ACSY01000090	Leu	CUG/CAG	Yes	47059-47141	No
cont1. 212	ACSY01000212	His	CAC/GTG	Yes	27634-27564	No
cont1. 245	ACSY01000245	Thr	ACA/TGT	No	23617-23689	No
cont1. 275	ACSY01000275	Arg	CGA/TGT	No	11978-12050	No
cont1. 318	ACSY01000318	Ser	UCU/AGA	Yes	1659-1740	No
cont1. 318	ACSY01000318	Ser	UCU/AGA	Yes	8388-8307	No
cont1. 328	ACSY01000328	Ser	UCU/AGA	Yes	5084-5003	No
cont1. 367	ACSY01000367	Thr	ACA/TGT	No	812-907	No
cont1. 367	ACSY01000367	Thr	ACA/TGT	No	1825-1920	No
cont1. 367	ACSY01000367	Ala	GCU/AGC	No	16335-16263	No
cont1. 397	ACSY01000397	Leu	CUG/CAG	Yes	3110-3027	No
cont1. 397	ACSY01000397	Gln	CAG/CTG	Yes	5303-5232	No
cont1.452	ACSY01000452	Ser	UCA/TGA	No	363-260	No
cont1.457	ACSY01000457	Tyr	UAC/GTA	Yes	107056-106969	No
cont1.459	ACSY01000459	Tyr	UAC/GTA	Yes	1860-1950	No
cont1.484	ACSY01000484	Tyr	UAC/GTA	Yes	36383-36294	No

cont1.489	ACSY01000489	Glu	GAG/CTC	Yes	69217-69146	No
cont1.489	ACSY01000489	Glu	GAG/CTC	Yes	75372-75443	No
cont1.505	ACSY01000505	Lys	AAA/TTT	No	8378-8275	No
cont1.655	ACSY01000655	Lys	AAG/CTT	Yes	44629-44555	No
cont1.655	ACSY01000655	Lys	AAG/CTT	Yes	45387-45313	No
cont1.655	ACSY01000655	Lys	AAG/CTT	Yes	52615-52689	No
cont1.666	ACSY01000666	Lys	AAG/CTT	Yes	31652-31578	No
cont1.666	ACSY01000666	Lys	AAG/CTT	Yes	33653-33727	No
cont1.666	ACSY01000666	Lys	AAG/CTT	Yes	34397-34471	No
cont1.680	ACSY01000680	Glu	GAG/CTC	Yes	26489-26418	No
cont1.680	ACSY01000680	Val	GUA/TAC	No	31401-31473	No
cont1.683	ACSY01000683	Glu	GAG/CTC	Yes	2063-1992	No
cont1.726	ACSY01000726	Asn	AAC/GTT	Yes	3434-3507	No
cont1.782	ACSY01000782	Met	AUG/CAT	n/a	7213-7142	No
cont1.782	ACSY01000782	Met	AUG/CAT	n/a	7429-7358	No
cont1.807	ACSY01000807	Phe	UUC/GAA	Yes	40327-40253	Yes
cont1.807	ACSY01000807	Phe	UUC/GAA	Yes	56155-56229	Yes
cont1.915	ACSY01000915	Pro	CCU/AGG	No	8498-8427	No
cont1.921	ACSY01000921	Thr	ACG/CGT	Yes	842-914	No
cont1.921	ACSY01000921	Thr	ACG/CGT	Yes	12059-11987	No
cont1.957	ACSY01000957	Cys	UGC/GCA	Yes	6143-6214	No
cont1.958	ACSY01000958	Leu	UUA/TAA	No	54251-54322	No
cont1.984	ACSY01000984	Gly	GGA/TCC	No	26963-26872	No
cont1.1036	ACSY01001036	Cys	UGC/GCA	Yes	20302-20374	No
cont1.1077	ACSY01001077	Phe	UUC/GAA	Yes	125-52	Yes
cont1.1078	ACSY01001078	Phe	UUC/GAA	Yes	2193-2285	Yes

cont1.1104	ACSY01001104	Ala	GCG/CGC	No	60799-60728	No
cont1.1107	ACSY01001107	Ala	GCU/TGC	No	2346-2417	No
cont1.1110	ACSY01001110	Ser	AGC/GCT	Yes	3878-3805	No
cont1.1111	ACSY01001111	Ala	GCG/CGC	No	15830-15901	No
cont1.1111	ACSY01001111	Ala	GCU/TGC	No	11758-11687	No
cont1.1124	ACSY01001124	Asp	GAC/GTC	Yes	1307-1378	No
cont1.1125	ACSY01001125	Asp	GAC/GTC	Yes	588-659	No
cont1.1128	ACSY01001128	Asp	GAC/GTC	Yes	26964-26893	No
cont1.1130	ACSY01001130	Asp	GAC/GTC	Yes	1106-1035	No
cont1.1175	ACSY01001175	Ile	AUU/AAT	No	7977-7904	No
cont1.1305	ACSY01001305	Pro	CCG/CGG	Yes	652-581	No
cont1.1333	ACSY01001333	Leu	CUU/AAG	No	57342_57424	No
cont1.1333	ACSY01001333	Leu	CUU/AAG	No	63168-63086	No
cont1.1382	ACSY01001382	Leu	UUG/CAA	No	596-679	No
cont1.1410	ACSY01001410	His	CAC/ GTG	Yes	5088-5018	No
cont1.1410	ACSY01001410	His	CAC/ GTG	Yes	11633-11703	No
cont1.1477	ACSY01001477	Tyr	TAC/GTA	Yes	1939-1852	No
cont1.1493	ACSY01001493	Arg	CGU/ACG	No	74281-74354	No
cont1.1503	ACSY01001503	Pro	CCU/AGG	No	16500-16571	No
cont1.1503	ACSY01001503	Pro	CCU/AGG	No	17645-17574	No
cont1.1515	ACSY01001515	Arg	CGU/ACG	No	74281-74354	No
cont1.1515	ACSY01001515	Arg	CGU/ACG	No	86724-86651	No
cont1.1530	ACSY01001530	Met	AUG/CAT	n/a	555-448	No
cont1.1569	ACSY01001569	Met	AUG/CAT	n/a	7721-7648	No
cont1.1584	ACSY01001584	Val	GUU/AAC	No	40020-39947	No
cont1.1588	ACSY01001588	Val	GUU/AAC	No	84722-84795	No

cont1.1594	ACSY01001594	Val	GUG/CAC	No	28055-28128	No
cont1.1594	ACSY01001594	Val	GUG/CAC	No	36114-36041	No
cont1.1603	ACSY01001603	Val	GUU/AAC	No	5498-5571	No
cont1.1603	ACSY01001603	Val	GUU/AAC	No	5689-5762	No
cont1.1605	ACSY01001605	Val	GUG/CAC	No	303-381	No
cont1.1780	ACSY01001780	Arg	CGG/CCG	No	15213-15140	No
cont1.1910	ACSY01001910	Ala	GCU/AGC	No	10887-10815	No
cont1.1910	ACSY01001910	Ala	GCU/AGC	No	16705-16777	No
cont1.1997	ACSY01001997	Glu	GAA/TTC	No	418-345	No
cont1.2001	ACSY01002001	Glu	GAA/TTC	No	1016-1089	No
cont1.2077	ACSY01002077	Trp	TGG/CCA	n/a	10963-11035	No
cont1.2077	ACSY01002077	Trp	TGG/CCA	n/a	16108-16036	No
cont1.2180	ACSY01002180	Asp	GAC/GTC	Yes	31575-31646	No
cont1.2180	ACSY01002180	Asp	GAC/GTC	Yes	34471-34542	No
cont1.2198	ACSY01002198	Arg	CGU/ACG	No	8914-8841	No
cont1.2214	ACSY01002214	Arg	CGU/ACG	No	4060-3987	No
cont1.2233	ACSY01002233	Leu	CUU/AAG	No	1026-944	No
cont1.2236	ACSY01002236	Leu	CUU/AAG	No	711-793	No
cont1.2238	ACSY01002238	Ser	UCG/CGA	Yes	59-143	Yes
cont1.2258	ACSY01002258	Pro	CCA/TGG	No	3477-3406	No
cont1.2258	ACSY01002258	Pro	CCA/TGG	No	10766-10837	No
cont1.2303	ACSY01002303	Gly	GGG/CCC	No	59349-59420	No
cont1.2378	ACSY01002378	Gly	GGC/GCC	Yes	19894-20000	No
cont1.2380	ACSY01002380	Asn	AAC/GTT	Yes	417-490	No
cont1.2380	ACSY01002380	Gly	GGC/GCC	Yes	667-560	No
cont1.2380	ACSY01002380	Gly	GGC/GCC	Yes	866-760	No

cont1.2566	ACSY01002566	Gln	CAG/CTG	Yes	100533-100462	No
cont1.2566	ACSY01002566	Gln	CAG/CTG	Yes	100873-100944	No
cont1.2660	ACSY01002660	Gly	GGC/GCC	Yes	1946-2052	No
cont1.2660	ACSY01002660	Gly	GGC/GCC	Yes	2146-2253	No
cont1.2660	ACSY01002660	Asn	AAC/GTT	Yes	2394-2321	No
cont1.2664	ACSY01002664	Gly	GGC/GCC	Yes	1699-1593	No
cont1.2664	ACSY01002664	Gly	GGC/GCC	Yes	7530-7636	No
cont1.2665	ACSY01002665	Asn	AAC/GTT	Yes	8488-8561	No
cont1.2683	ACSY01002683	Tyr	TAC/GTA	Yes	13537-13624	No
cont1.2714	ACSY01002714	Ser	AGC/GCT	Yes	1591-1515	No
cont1.2788	ACSY01002788	Arg	AGG/CCT	No	19894-19976	No
cont1.2856	ACSY01002856	Ala	GCU/AGC	No	1286-1214	No
cont1.2857	ACSY01002857	Leu	CUA/TAG	No	14752-14834	No
cont1.2857	ACSY01002857	Ala	GCU/AGC	No	76148-76220	No
cont1.2966	ACSY01002966	Met	AUG/CAT	n/a	1068-961	No
cont1.2966	ACSY01002966	Met	AUG/CAT	n/a	6245-6352	No

S4c. Predicted *C. owczarzaki* tRNA genes.

Scaffold	Accession Number	tRNA Gene	Codon/tRNA Anti-codon	Codonw Optimal Codon	Co-ordinates	Putative Pseudogene
cont2.2	ACFS02000002	Ala	GCU/AGC	No	205973-206045	No
cont2.2	ACFS02000002	Gln	CAG/CTG	Yes	206261-206339	No
cont2.2	ACFS02000002	Gln	CAG/CTG	Yes	206555-206633	No
cont2.2	ACFS02000002	Ala	GCU/AGC	No	210968-211040	No
cont2.5	ACFS02000005	Glu	GAG/CTC	Yes	23594-23522	No
cont2.5	ACFS02000005	Glu	GAG/CTC	Yes	23866-23794	No
cont2.5	ACFS02000005	Glu	GAG/CTC	Yes	24086-24158	No
cont2.5	ACFS02000005	Glu	GAA/TTC	No	24341-24413	No
cont2.5	ACFS02000005	Glu	GAA/TTC	No	24501-24573	No
cont2.6	ACFS02000006	Glu	GAG/CTC	Yes	9138-9066	No
cont2.13	ACFS02000013	Met	ATG/CAT	n/a	63011-62939	Yes
cont2.13	ACFS02000013	Met	ATG/CAT	n/a	67578-67650	Yes
cont2.15	ACFS02000015	Val	GUA/TAC	No	53953-54025	No
cont2.17	ACFS02000017	Arg	AGG/CCT	No	68149-68078	No
cont2.32	ACFS02000032	Ile	AUU/TAA	No	137249-137176	No
cont2.32	ACFS02000032	Leu	CUU/AAG	No	289203-289121	No
cont2.45	ACFS02000045	Ala	GCU/AGC	No	137746-137674	No
cont2.58	ACFS02000058	Leu	CUA/TAG	No	9611-9531	Yes
cont2.66	ACFS02000066	Val	GUU/AAC	No	66048-65976	No
cont2.68	ACFS02000068	Gln	GAA/TTC	No	36549-36478	No
cont2.73	ACFS02000073	Pro	CCU/AGG	No	26057-26128	No
cont2.75	ACFS02000075	Pro	CCU/AGG	No	24367-24296	No

cont2.75	ACFS02000075	Pro	CCU/AGG	No	24539-24468	No
cont2.75	ACFS02000075	Thr	ACU/AGT	No	290002-290074	No
cont2.83	ACFS02000083	Trp	TGG/CCA	n/a	55092-55020	No
cont2.95	ACFS02000095	Ala	GCA/TGC	No	36684-36753	No
cont2.95	ACFS02000095	Ala	GCA/TGC	No	36844-36915	No
cont2.111	ACFS02000111	Gly	GGC/GCC	Yes	121275-121345	No
cont2.111	ACFS02000111	Leu	UUG/CAA	No	219295-219214	No
cont2.114	ACFS02000114	Ser	AGC/GCT	No	9138-9053	No
cont2.118	ACFS02000118	His	CAC/GTG	Yes	48971-49041	No
cont2.118	ACFS02000118	His	CAC/GTG	Yes	49137-49207	No
cont2.119	ACFS02000119	His	CAC/GTG	Yes	120052-120122	No
cont2.120	ACFS02000120	Ser	UCU/AGA	Yes	6336-6443	No
cont2.120	ACFS02000120	Ser	UCU/AGA	Yes	6657-6764	No
cont2.120	ACFS02000120	Ser	UCG/CGA	Yes	7055-6964	No
cont2.120	ACFS02000120	Ser	UCG/CGA	Yes	8597-8688	No
cont2.122	ACFS02000122	Arg	CGU/ACG	Yes	29064-29136	No
cont2.124	ACFS02000124	Ser	AGC/GCT	No	127323-127239	No
cont2.147	ACFS02000147	Ser	UCA/TGA	No	71475-71393	No
cont2.148	ACFS02000148	Pro	CCA/TGG	No	71378-71453	No
cont2.150	ACFS02000150	Ile	AUU/AAT	No	52208-52281	No
cont2.150	ACFS02000150	Thr	ACU/AGT	No	101052-100980	No
cont2.150	ACFS02000150	Trp	TGG/CCA	n/a	107514-107586	No
cont2.155	ACFS02000155	Asn	AAC/GTT	Yes	12240-12312	No
cont2.174	ACFS02000174	Cys	UGC/GCA	Yes	166788-166717	No
cont2.181	ACFS02000181	Lys	AAG/CTT	Yes	22396-22468	No
cont2.181	ACFS02000181	Lys	AAG/CTT	Yes	22624-22696	No

cont2.181	ACFS02000181	Lys	AAG/CTT	Yes	25061-24989	No
cont2.181	ACFS02000181	Lys	AAG/CTT	Yes	41589-41661	No
cont2.181	ACFS02000181	Val	GUG/CAC	No	96468-96397	No
cont2.183	ACFS02000183	Asn	AAC/GTT	Yes	95586-95658	No
cont2.183	ACFS02000183	Asn	AAC/GTT	Yes	95763-95835	No
cont2.183	ACFS02000183	Thr	ACG/CGT	No	106876-106804	No
cont2.185	ACFS02000185	Thr	ACU/AGT	No	2041-1969	No
cont2.185	ACFS02000185	Thr	ACG/CGT	No	2213-2141	No
cont2.191	ACFS02000191	Pro	CCG/CGG	No	129600-129671	No
cont2.191	ACFS02000191	Pro	CCG/CGG	No	129746-129817	No
cont2.193	ACFS02000193	Gly	GGA/TCC	No	84593-84663	No
cont2.197	ACFS02000197	Ser	UCU/AGA	Yes	57098-56991	No
cont2.207	ACFS02000207	Asp	GAC/GTC	Yes	4350-4421	Yes
cont2.207	ACFS02000207	Leu	CUG/CAG	No	52992-52912	No
cont2.209	ACFS02000209	Val	GUU/AAC	No	182494-182566	No
cont2.209	ACFS02000209	Val	GUU/AAC	No	182583-182655	No
cont2.214	ACFS02000214	Met	AUG/CAT	n/a	21542-21614	No
cont2.217	ACFS02000217	Leu	UUA/TAA	No	6754 -6672	No
cont2.230	ACFS02000230	Asp	GAC/GTC	Yes	46985-46914	Yes
cont2.238	ACFS02000238	Arg	CGU/ACG	Yes	14090-14162	No
cont2.238	ACFS02000238	Arg	CGU/ACG	Yes	16021-16093	No
cont2.239	ACFS02000239	Gly	GGC/GCC	Yes	10374-10304	No
cont2.259	ACFS02000259	Gly	GGC/GCC	Yes	40625-40695	No
cont2.265	ACFS02000265	Cys	UGC/GCA	Yes	132739-132668	No
cont2.285	ACFS02000285	Ile	AUU/AAT	No	27587-27514	No
cont2.285	ACFS02000285	Ile	AUU/AAT	No	27687-27760	No

cont2.294	ACFS02000294	Phe	UUC/GAA	Yes	251261-251333	No
cont2.294	ACFS02000294	Phe	UUC/GAA	Yes	251596-251524	No
cont2.294	ACFS02000294	Phe	UUC/GAA	Yes	251766-251694	No
cont2.301	ACFS02000301	Ala	GCU/AGC	No	1244-1172	No
cont2.310	ACFS02000310	Tyr	UAC/GTA	Yes	26461-26377	No
cont2.310	ACFS02000310	Arg	AGA/TCT	No	86962-86875	No
cont2.314	ACFS02000314	Gly	GGC/GCC	Yes	144337-144267	No
cont2.326	ACFS02000326	Glu	GAG/CTC	Yes	13234-13162	No
cont2.330	ACFS02000330	Arg	CGU/TCG	Yes	19806-19876	No
cont2.331	ACFS02000331	Val	GUU/AAC	No	13581-13509	No
cont2.351	ACFS02000351	Met	AUG/CAT	n/a	95311-95240	No
cont2.351	ACFS02000351	Met	AUG/CAT	n/a	95500-95429	No
cont2.356	ACFS02000356	SeC	UGA/TCA	n/a	2249-2336	No
cont2.370	ACFS02000370	Lys	AAG/CTT	Yes	125067-124995	No
cont2.372	ACFS02000372	Lys	AAG/CTT	Yes	99846-99774	No
cont2.375	ACFS02000375	Asp	GAC/GTC	Yes	173301-173230	No
cont2.379	ACFS02000379	Ala	GCU/AGC	No	33933-34005	No
cont2.387	ACFS02000387	Leu	CUG/CAG	No	18867-18787	No
cont2.411	ACFS02000411	Ala	GCU/AGC	No	23235-23307	No
cont2.423	ACFS02000423	Ala	GCG/CGC	No	28556-28484	No
cont2.423	ACFS02000423	Tyr	UAC/GTA	Yes	22356-22272	No
cont2.426	ACFS02000426	Thr	ACA/TGT	No	47395-47498	No
cont2.427	ACFS02000427	Arg	CGU/ACG	Yes	1590-1518	No
cont2.430	ACFS02000430	Und	NNN	n/a	4307-4377	No
cont2.430	ACFS02000430	Gly	GGA/TCC	No	4379-4449	No
cont2.434	ACFS02000434	Gly	GGC/GCC	Yes	143428-14349	No

cont2.434	ACFS02000434	Gly	GGC/GCC	Yes	143186-14311	No
cont2.439	ACFS02000439	Ala	GCG/CGC	No	341298-34122	No
cont2.439	ACFS02000439	Gln	CAG/CTG	Yes	205604-20553	No
cont2.444	ACFS02000444	Thr	ACU/AGT	No	13551-13623	No
cont2.449	ACFS02000449	Leu	CUU/AAG	No	2646-2728	No
cont2.451	ACFS02000451	Asp	GAC/GTC	Yes	130398-13032	Yes
cont2.452	ACFS02000452	Asp	GAC/GTC	Yes	1942-2013	Yes
cont2.452	ACFS02000452	Asp	GAC/GTC	Yes	1721-1650	Yes
cont2.471	ACFS02000471	Leu	CUU/AAG	No	13262-13344	No
cont2.498	ACFS02000498	Gly	GGA/TCC	No	22-92	No
cont2.498	ACFS02000498	Asp	GAC/GTC	Yes	94-164	No
cont2.498	ACFS02000498	His	CAC/GTG	Yes	167-237	No
cont2.521	ACFS02000521	Leu	CUU/AAG	No	5125-5207	No

Table S5. Optimal codons which lack perfectly matching complementary tRNA genes.

**Amino Acid**	***M. brevicollis***	***S. rosetta***	***C. owczarzaki***
**Leu**	CUC	CUC	CUC
**Ile**	AUC	AUC	AUC
**Val**	GUC	GUC	GUC
**Ser**	UCC	UCC	UCC
**Pro**	CCC	CCC	CCC
**Thr**	ACC	ACC	ACC
**Ala**	GCC	GCC	GCC
**Arg**	CGC	CGC	CGC
**Gly**	GGU	-	-

Table S6. Presence and absence of modified tRNA molecules in transcriptome datasets.

**Amino Acid**	**tRNA Molecule**	**Sequence**[b]	**Transcriptome**
S. rosetta[a]			
**Leu**	tRNA^{Leu}_{AAG}	GATCAGGTGGCCGAGTTGGTCTAAGGCGTGCGGTTAAGGCCCGCATCTCTTCGGAGGCGCGAGTTCGAATCTCGTCCTGATCA	Absent
	$\text{tRNA}^{Leu}_{\mathbf{G}AG}$	GATCAGGTGGCCGAGTTGGTCTAAGGCGTGCGGTT**G**AGGCCCGCATCTCTTCGGAGGCGCGAGTTCGAATCTCGTCCTGATCA	Absent
**Ile**	tRNA^{Ile}_{AAT}	GCTCCATTGGCGCAGTTGGTTAGCGCGTGAGTCTAATAAACTCAAGGTCATCAGTTCGAGCCTGGTATGGAGCA	**Present**
	$\text{tRNA}^{Ile}_{\mathbf{G}AT}$	GCTCCATTGGCGCAGTTGGTTAGCGCGTGAGTCT**G**ATAAACTCAAGGTCATCAGTTCGAGCCTGGTATGGAGCA	**Present**
**Val**	tRNA^{Val}_{AAC}	GTTTCCGTGGTGTAGTTGGCTATCACATCAGTCTAACACACTGAAGGTCTCCGGTTCGAGCCCGGACGGAAACA	Absent
	$\text{tRNA}^{Val}_{\mathbf{G}AC}$	GTTTCCGTGGTGTAGTTGGCTATCACATCAGTCT**G**ACACACTGAAGGTCTCCGGTTCGAGCCCGGACGGAAACA	Absent
**Ser**	tRNA^{Ser}_{AGA}	GCACTCATGGCCGAGTGGTTAAGGCGAACGACTAGAAATCGTTTGGGCTCTGCCCGCACAGGTTCGAATCCTGTTGGGTGCG	**Present**
	$\text{tRNA}^{Ser}_{\mathbf{G}GA}$	GCACTCATGGCCGAGTGGTTAAGGCGAACGACT**G**GAAATCGTTTGGGCTCTGCCCGCACAGGTTCGAATCCTGTTGGGTGCG	**Present**
**Pro**	tRNA^{Pro}_{AGG}	GGTCCTGTGGTCTAGGGGTATGATTTCCGCTTAGGGTGCGGGAGGTCCCGGGTTCAAATCCCGGCAGGACCC	**Present**
	$\text{tRNA}^{Pro}_{\mathbf{G}GG}$	GGTCCTGTGGTCTAGGGGTATGATTTCCGCTT**G**GGGTGCGGGAGGTCCCGGGTTCAAATCCCGGCAGGACCC	**Present**
**Ala**	tRNA^{Ala}_{AGC}	GGGGCTGTAGCTCAAGTGGTAGAGCGCTCGCTTAGCATGCGAGAGGTACCGGGATCGATACCCGGCAGCTCCA	Absent
	$\text{tRNA}^{Ala}_{\mathbf{G}GC}$	GGGGCTGTAGCTCAAGTGGTAGAGCGCTCGCTT**G**GCATGCGAGAGGTACCGGGATCGATACCCGGCAGCTCCA	**Present**
**Arg**	tRNA^{Arg}_{ACG}	GGCCGTGTGGCCCAATTGGATAAGGCGCCTGACTACGGATCAGGAGATTCGGGGTTCGAGTCCCTGCATGGTCA	Absent
	$\text{tRNA}^{Arg}_{\mathbf{G}CG}$	GGCCGTGTGGCCCAATTGGATAAGGCGCCTGACTGCGGATCAGGAGATTCGGGGTTCGAGTCCCTGCATGGTCA	Absent
C. owczarzaki			
**Leu**	tRNA^{Leu}_{AAG}	GTGGTTGTGGCGGAGCTGGTCTAACGCGTCAGATTAAGGCTCTGATCACTTCGGTGGCCTGGGTTCGAATCCCAGCAACCACA	Absent
	$\text{tRNA}^{Leu}_{\mathbf{G}AG}$	GTGGTTGTGGCGGAGCTGGTCTAACGCGTCAGATT**G**AGGCTCTGATCACTTCGGTGGCCTGGGTTCGAATCCCAGCAACCACA	Absent
**Ile**	tRNA^{Ile}_{AAT}	GCTCGATTGGCACAGTTGGTTAGCGCGTGGTGCTAATAACGCCACGGTCGCAGGTTCGATCCCTGCATCGAGCA	Absent
	$\text{tRNA}^{Ile}_{\mathbf{G}AT}$	GCTCGATTGGCACAGTTGGTTAGCGCGTGGTGCT**G**ATAACGCCACGGTCGCAGGTTCGATCCCTGCATCGAGCA	Absent
**Val**	tRNA^{Val}_{AAC}	GGGCGTCTAGCTCATTTGGTAGAGCATGTCGTTAACACCGACAAGGTACGGAGATCGAAACTCTGGATGCTCA	Absent
	$\text{tRNA}^{Val}_{\mathbf{G}AC}$	GGGCGTCTAGCTCATTTGGTAGAGCATGTCGTT**G**ACACCGACAAGGTACGGAGATCGAAACTCTGGATGCTCA	Absent
**Ser**	tRNA^{Ser}_{AGA}	GGGCTGTTGGGTGAGCGGTTAAGCCGGTTGACTAGAAAGGTCTTTGTAATGAAGATCCTAATTATCAACTCCGATTTTCGGGCAGAGGTTCGAGTCCTCTA<br>CAGTCCG	Absent
	tRNA^{Ser}_{AGA}	GGGCTGTTGGGTGAGCGGTTAAGCCGGTTGACTAGAATGGTCTTCTCAACGAAGATCCTGATTATCAACTCCGATTTTCGGGCAGAGGTTCGAGTCCTCTA<br>CAGTCCG	Absent
	tRNA^{Ser}_{AGA}	GGGTTGTTGGGTGAGCGGTTAAGCCGGTTGACTAGAAAGACCTTCGTAATGAAGCTCCTATGTATCAACTCCGATTTTCGGGCAGAGGTTCGAGTCCTCTA<br>CAATCCG	Absent
	$\text{tRNA}^{Ser}_{\mathbf{G}GA}$	GGGCTGTTGGGTGAGCGGTTAAGCCGGTTGACT**G**GAAAGGTCTTTGTAATGAAGATCCTAATTATCAACTCCGATTTTCGGGCAGAGGTTCGAGTCCTCTA<br>CAGTCCG	Absent

	$\text{tRNA}^{\text{Ser}}_{\mathbf{G}\text{GA}}$	GGGCTGTTGGGTGAGCGGTTAAGCCGGTTGACT**G**GAATGGTCTTCTCAACGAAGATCCTGATTATCAACTCCGATTTTCGGGCAGAGGTTCGAGTCCTCTACAGTCCG	Absent
	$\text{tRNA}^{\text{Ser}}_{\mathbf{G}\text{GA}}$	GGGTTGTTGGGTGAGCGGTTAAGCCGGTTGACT**G**GAAAGACCTTCGTAATGAAGCTCCTATGTATCAACTCCGATTTTCGGGCAGAGGTTCGAGTCCTCTACAATCCG	Absent
**Pro**	$\text{tRNA}^{\text{Pro}}_{\text{AGG}}$	GAGCTCGTAGTATAGTGGTATTATGCTCGCTTAGGGTGCGAGAGGTCGTGGGTTCAATTCCCGCCGAGCTCC	Absent
	$\text{tRNA}^{\text{Pro}}_{\mathbf{G}\text{GG}}$	GAGCTCGTAGTATAGTGGTATTATGCTCGCTT**G**GGGTGCGAGAGGTCGTGGGTTCAATTCCCGCCGAGCTCC	Absent
**Thr**	$\text{tRNA}^{\text{Thr}}_{\text{AGT}}$	GCTCCCATAGCTCAATTGGCAGAGCGCGACACTAGTAATGTCGAGGTAGCGTGTTCGATTCACGCTGGGTGCA	Absent
	$\text{tRNA}^{\text{Thr}}_{\mathbf{G}\text{GT}}$	GCTCCCATAGCTCAATTGGCAGAGCGCGACACT**G**GTAATGTCGAGGTAGCGTGTTCGATTCACGCTGGGTGCA	Absent
**Ala**	$\text{tRNA}^{\text{Ala}}_{\text{AGT}}$	GGGCTTGTAGCTCATTTGGTAGAGCGCATCGTTAGCATCGATGAGGTCAGCGGTTCGACTCCGTTCAAGTCCA	Absent
	$\text{tRNA}^{\text{Ala}}_{\mathbf{G}\text{GT}}$	GGGCTTGTAGCTCATTTGGTAGAGCGCATCGTT**G**GCATCGATGAGGTCAGCGGTTCGACTCCGTTCAAGTCCA	Absent
**Arg**	$\text{tRNA}^{\text{Arg}}_{\text{ACG}}$	GGTGCTATAGCACAGTTGGTAGTGCGTCTGGCTACGAACCAGGAGGTCGCAGGTTCGAGCCCTGCTAGCATCG	**Present**
	$\text{tRNA}^{\text{Arg}}_{\mathbf{G}\text{CG}}$	GGTGCTATAGCACAGTTGGTAGTGCGTCTGGCT**G**CGAACCAGGAGGTCGCAGGTTCGAGCCCTGCTAGCATCG	**Present**

[a] The *S. rosetta* genome lacks a $\text{tRNA}^{\text{Thr}}_{\text{AGC}}$ gene.

[b] Predicted sequences of modified tRNA have guanine in place of adenine in the wobble position of the anticodon and are highlighted in bold.

Table S7. Mean F_{op} values for domain and non-domain codons for the three codon usage bias categories in each species.

Species	Domain Codons F_{op} (±sd)	Non-Domain Codons F_{op} (±sd)
M. brevicollis[a]		
High Biased	0.787±0.068	0.714±0.146
Mid Biased	0.576±0.047	0.537±0.059
Low Biased	0.519±0.062	0.471±0.066
S. rosetta[b]		
High Biased	0.751±0.068	0.716±0.125
Mid Biased	0.602±0.055	0.542±0.067
Low Biased	0.516±0.069	0.475±0.093
C. owczarzaki[b]		
High Biased	0.740±0.102	0.648±0.163
Mid Biased	0.506±0.062	0.457±0.062
Low Biased	0.420±0.069	0.380±0.073

a: *M. brevicollis* optimal codons were defined by the RSCU correspondence analysis

b: *S. rosetta* and *C. owczarzaki* optimal codons were defined by comparing codon frequencies in the 5% highest and 5% lowest expressed genes

Table S8. Odds ratios and their log values in twofold degenerate amino acids.

	M. brevicollis		***S. rosetta***		***C. owczarzaki***	
	**Odds Ratio**	**Ln (Odds Ratio)**	**Odds Ratio**	**Ln (Odds Ratio)**	**Odds Ratio**	**Ln (Odds Ratio)**
**Phe**	1.85	0.62	1.89	0.63	2.52	0.93
**Tyr**	3.75	1.32	6.45	1.86	8.59	2.15
**His**	2.64	0.97	5.25	1.66	12.03	2.49
**Gln**	2.86	1.05	11.48	2.44	3.57	1.27
**Asn**	6.26	1.83	12.65	2.54	26.39	3.27
**Lys**	13.59	2.61	14.95	2.70	55.84	4.02
**Asp**	1.79	0.58	12.66	2.54	4.29	1.46
**Glu**	2.34	0.85	8.83	2.18	4.98	1.61
**Cys**	11.14	2.41	9.72	2.27	29.72	3.39
