# Supplementary figures and images for: Patterns of Ancestral Animal Codon Usage Bias Revealed through Holozoan Protists
Source: Mol Biol Evol. 2018 Aug 29;35(10):2499–511. doi: 10.1093/molbev/msy157 (PMC6188563; doi:10.1093/molbev/msy157)

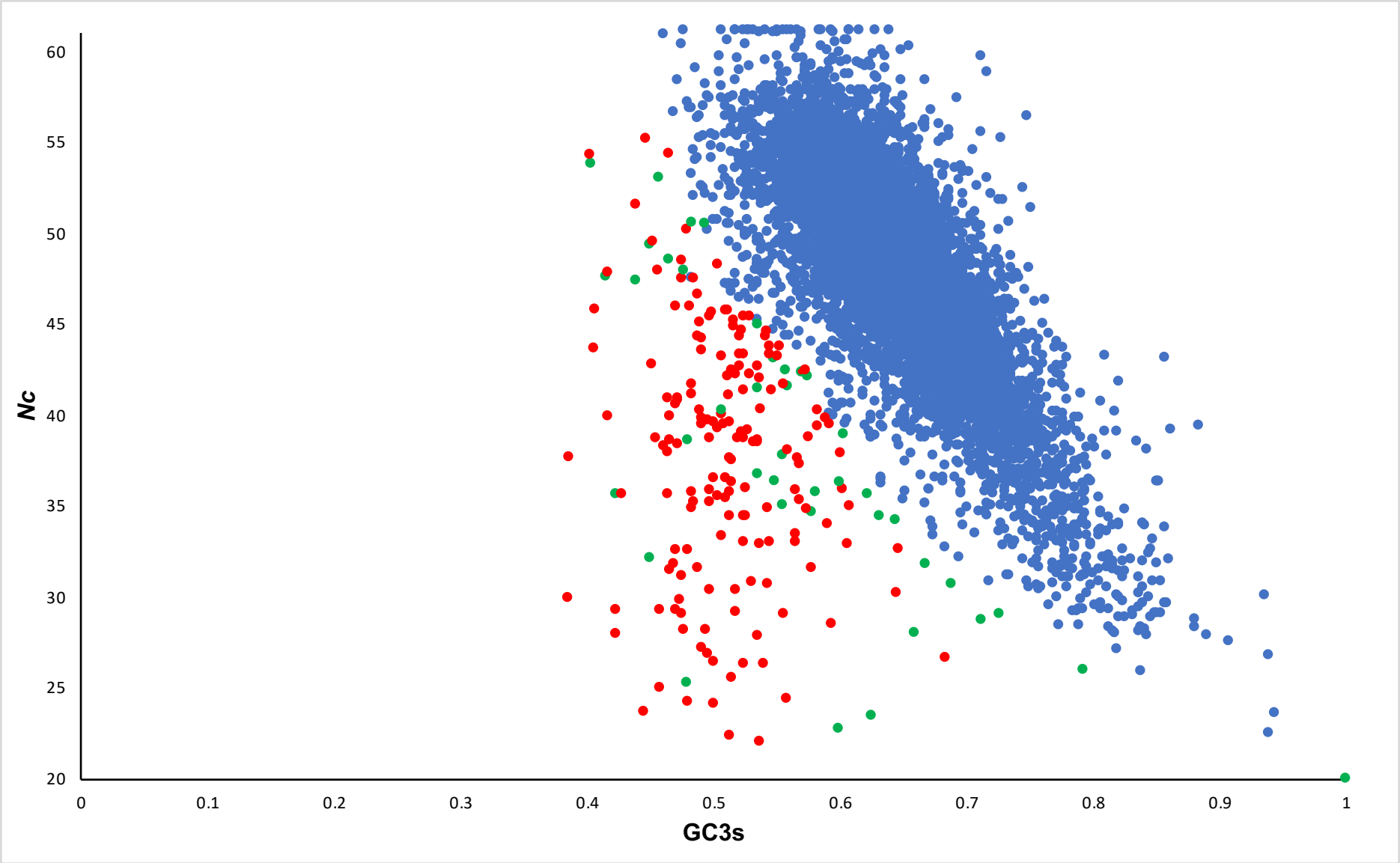

Supplement: Supplementary Data [file msy157_supp.zip › Figure S1.pdf]

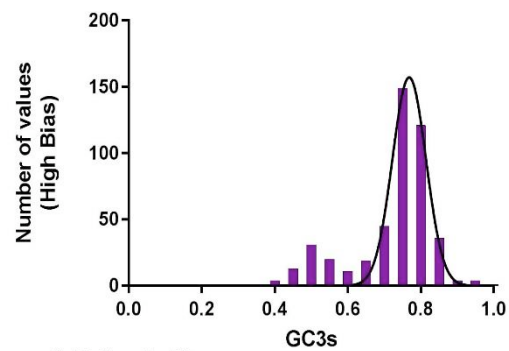

a) *M. brevicollis*

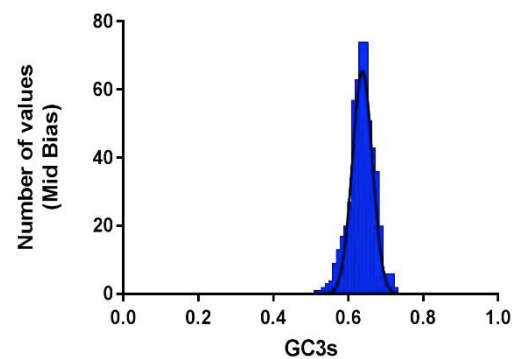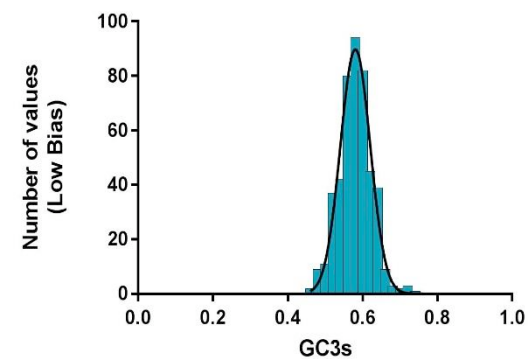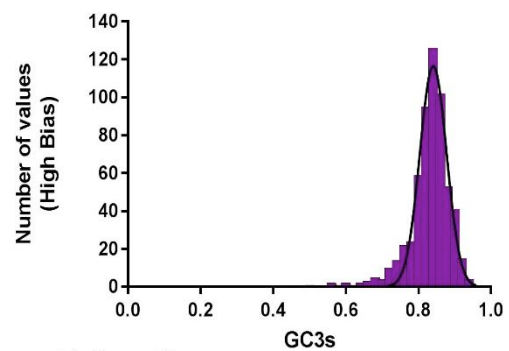

b) *S. rosetta*

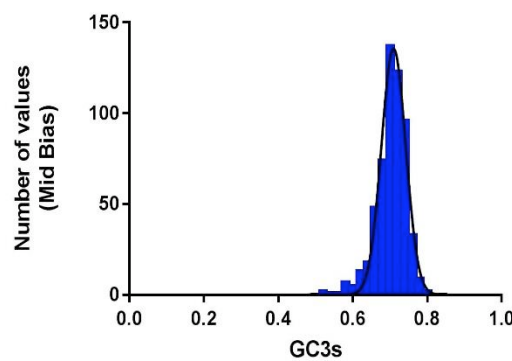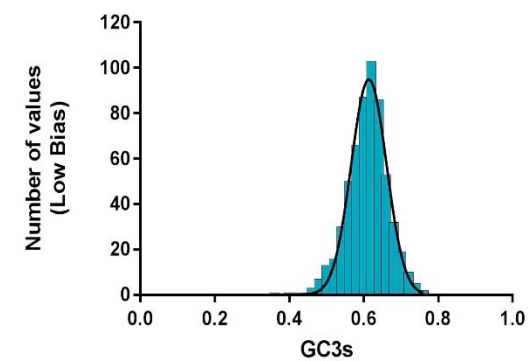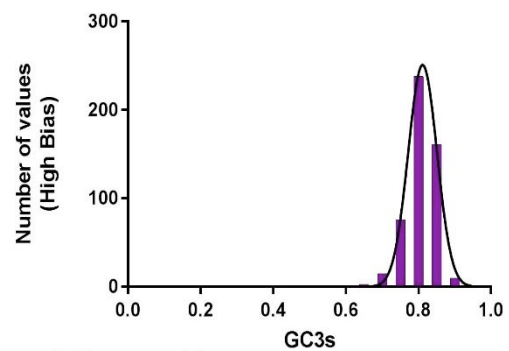

c) *C. owczarzaki*

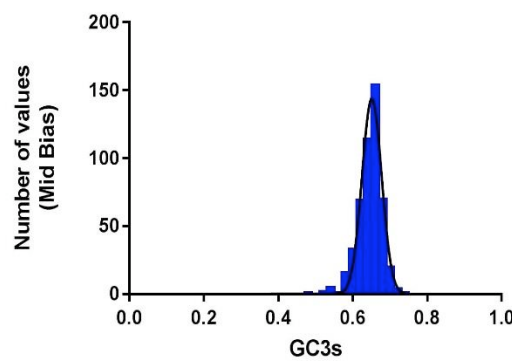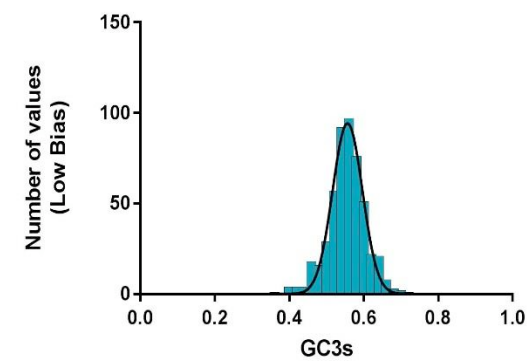

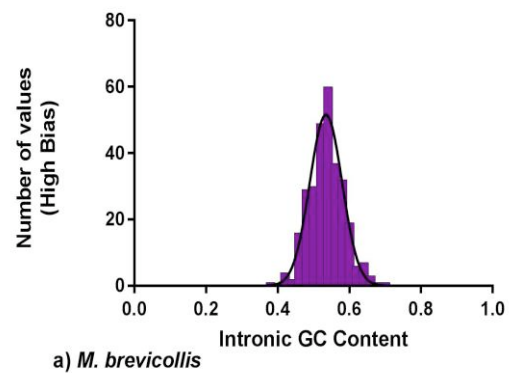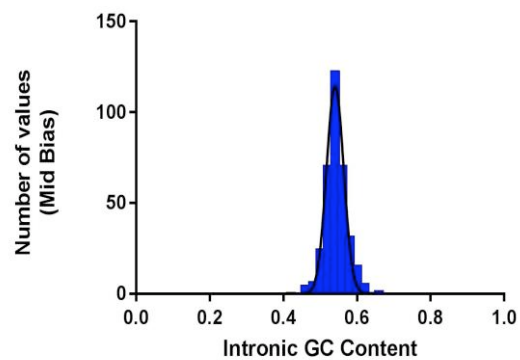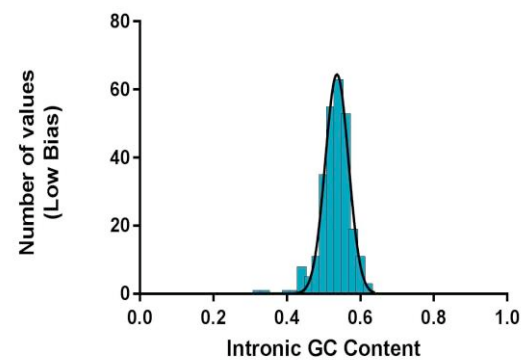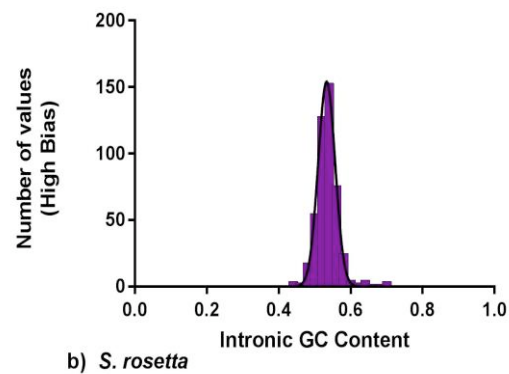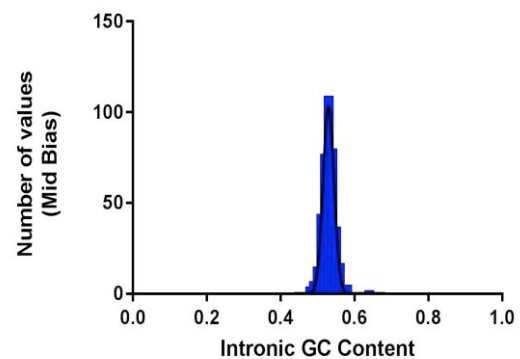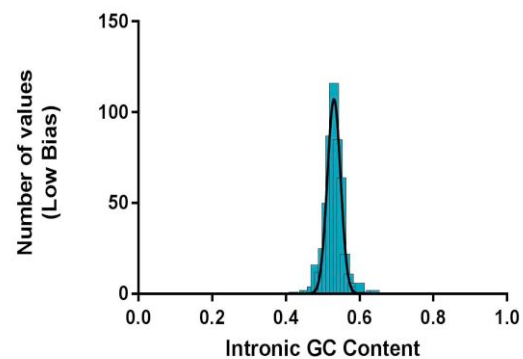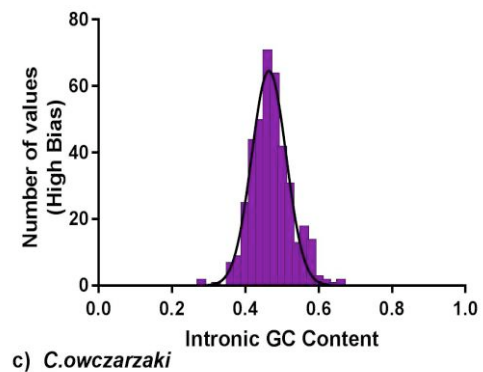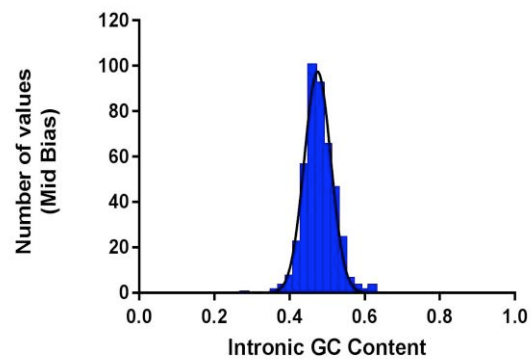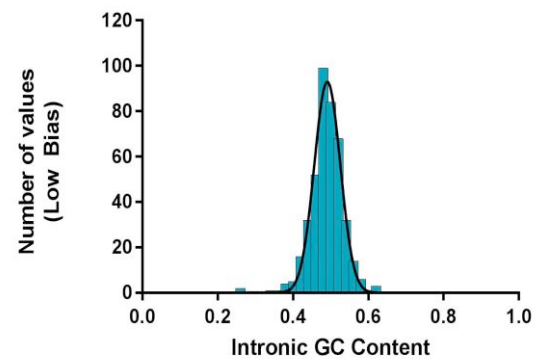

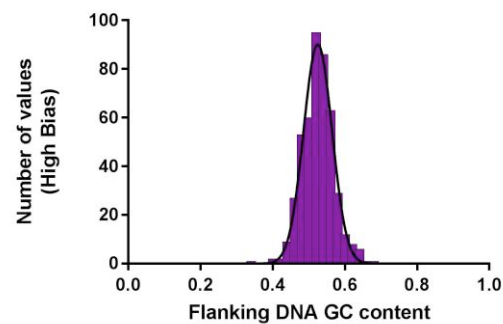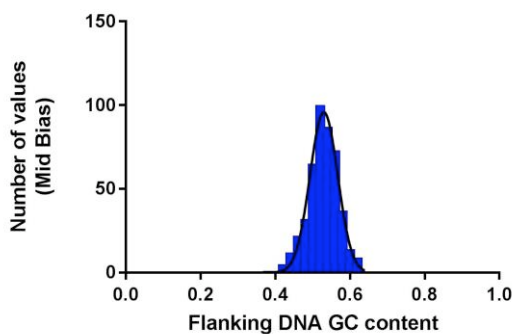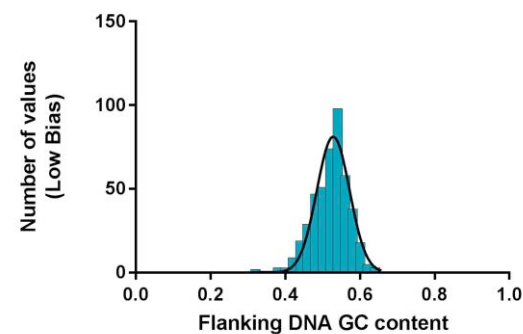

a) *M. brevicollis*

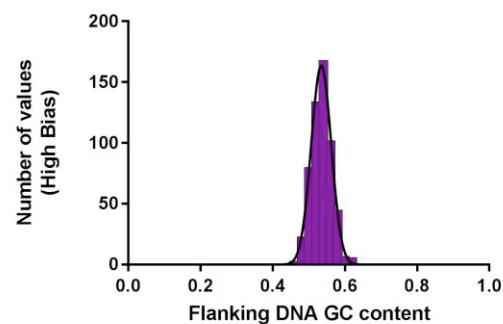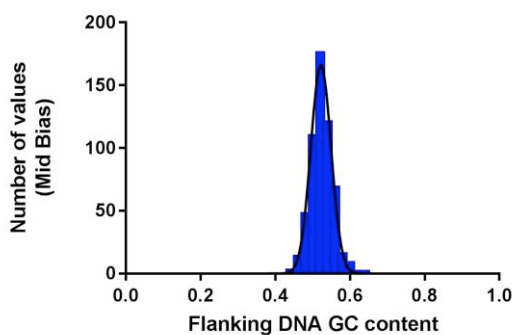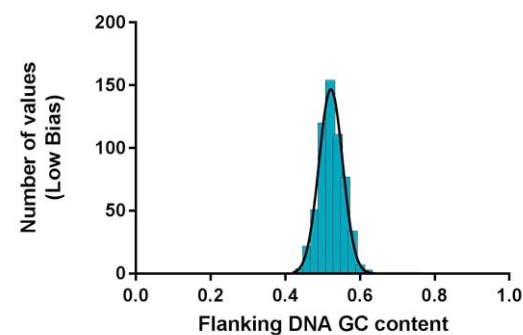

b) *S. rosetta*

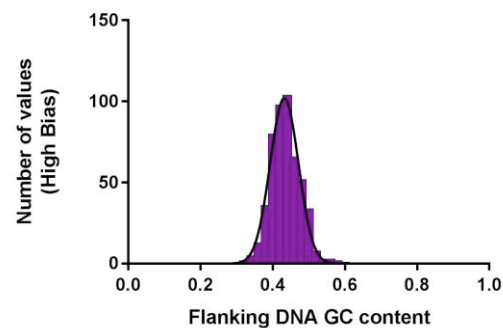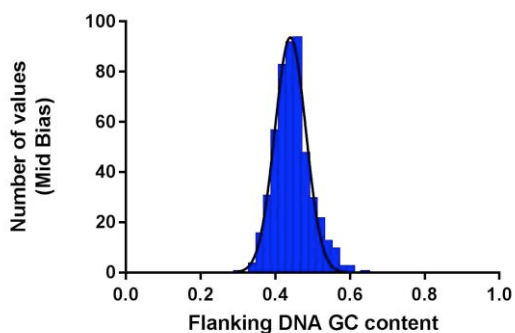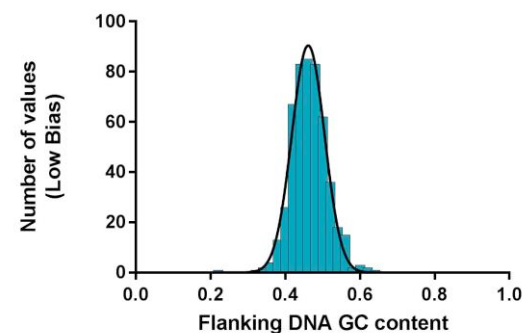

c) *C. owczarzaki*

Supplement: Supplementary Data [file msy157_supp.zip › Figure S2.pdf]
